# Prioritizing Sustainable Denim Fabric through Integrated Decision-Making Framework

**DOI:** 10.3390/ma17133291

**Published:** 2024-07-03

**Authors:** Eda Acar, Derya Tama Birkocak, Aşkın Özdağoğlu, Zümrüt Ünal, Gizem Özdemir, Maria Josè Abreu

**Affiliations:** 1 Textile Engineering Department, Ege University, 35040 İzmir, Türkiye; eda.acar@ege.edu.tr (E.A.); zumrut.bahadir.unal@ege.edu.tr (Z.Ü.); 2 Business Faculty, Dokuz Eylul University, Tınaztepe Yerleşkesi, 35390 İzmir, Türkiye; askin.ozdagoglu@deu.edu.tr; 3 Uniteks R&D Center, Atatürk OSB, 10039 Sk. No:26, 35620 İzmir, Türkiye; gizem.ozdemir@uniteks.com.tr; 4 2C2T—Centre for Textile Science and Technology, University of Minho, Campus de Azurém, 4800-058 Guimarães, Portugal; josi@det.uminho.pt

**Keywords:** denim fabric, recycled cotton, LMAW, DNMA, MCDM

## Abstract

In this study, a combined multi-criteria decision-making (MCDM) approach that integrates the logarithm methodology of additive weights (LMAW) and the double normalization-based multiple aggregation (DNMA) methods has been utilized to determine the optimal fabric structures considering the performance characteristics of denim fabrics containing recycled cotton. This approach focuses on sustainability and performance criteria, applying advanced decision-making methodologies to provide in-depth analysis and guidance for denim fabric selection. In this research, 15 distinct criteria were taken into account. Alternatives were ranked based on outcomes obtained from these methods. Although it was not anticipated that the top-ranked alternatives would simultaneously fulfill the beneficial or non-beneficial orientation of all criteria, an examination of the top three alternatives (A12, A5, and A15) for both garment groups revealed that they indeed aligned with the pre-determined criterion orientation. This highlights the effectiveness of the multi-criteria decision-making approach in the context of this study.

## 1. Introduction

Denim fabric, a distinctive and popular material in the fashion industry, is commonly used for making jeans, jackets, and other apparel. The global nature of denim fabric, is independent of age, gender, and economic factors, both in work and daily life. It is obtained by weaving blue indigo-dyed warp yarn with undyed weft yarn, typically featuring twill construction [1]. The global market for denim fabric, valued at approximately USD 27.1 billion in 2022, is expected to reach nearly USD 36 billion by 2027 [2]. This growth is driven by the versatility and popularity of denim, appealing to consumers of all ages.

The denim industry is transitioning towards sustainable production in response to increasing environmental awareness and regulations. Key strategies include the use of sustainable raw materials, circular economy practices, and energy-efficient technologies [3]. A significant challenge in denim production is the environmental impact, particularly due to the use of hazardous chemicals and greenhouse gas emissions. Manufacturers are focusing on reducing these impacts through sustainable practices, such as denim recycling [4].

Life cycle analyses, such as the one conducted by Levi Strauss & Co. (San Francisco, CA, USA) for their 501 jeans, reveal that substantial resources are still used in producing denim, including high water and energy consumption and chemical usage [1]. Levi Strauss & Co., in the life cycle assessment, studied the impacts of Levi’s 501 jeans on climate change and calculated carbon dioxide emissions throughout the life of jeans by cradle-to-grave approach. According to the results, the consumer phase emissions and fabric production phase are found to be the highest [5,6].

Denim jeans and jackets are the most extensive clothing in the entire globe, and their raw materials are almost made of 100% cotton fibers [7]. Cotton, the key raw material for the denim industry, has significant environmental impacts due to its water, land, energy, fertilizer, and pesticide usage. To mitigate these effects, the use of recycled cotton is becoming increasingly important [3,8,9]. Beyond cotton, other fibers like polyester, wool, elastane, and linen are being incorporated to meet diverse consumer demands, including adding flexibility to traditional denim fabrics.

Recycled cotton, derived from pre-consumer or post-consumer waste, helps reduce the environmental footprint of denim production. It conserves resources by minimizing the need for new raw materials and reduces waste. Life cycle analysis has shown that using recycled cotton in denim production results in lower environmental impact compared to conventional cotton [10].

The literature overview demonstrates the multifaceted nature of denim fabric research, encompassing sustainability, environmental impacts, and advanced methodologies for fabric optimization and assessment. The performance characteristics of denim fabrics produced from recycled cotton, such as mechanical properties and comfort, are critical in evaluating their suitability for various applications, and denim fabrics are evolving in these aspects [11,12]. The study by Alp et al. (2023) evaluated the effect of recycled cotton on the performance characteristics of denim fabrics produced from open-ended rotor weft yarns [13]. Sustainability in denim production encompasses various aspects, including sustainable washing [14,15,16,17,18], bleaching [19,20], dyeing, and finishing processes [21,22,23].

The incorporation of the versatile nature of denim fabric production has made performance evaluation a complex multi-criteria decision-making problem. Limited studies have applied MCDM approaches to evaluate recycled denim fabrics. Building upon the sustainability assessment in denim production using a multi-criterion decision-making (MCDM) technique, Fidan et al. (2021) conducted an integrated life cycle assessment for denim fabric production using recycled cotton fibers and a combined heat and power plant [3]. This study investigated eight different scenarios, applying the TODIM method to evaluate sustainability dimensions.

Yildirim et al. (2022) utilized the MULTIMOORA method, another MCDM approach, to select the most suitable yarn type for denim manufacturing among 27 different yarn types [24]. This highlights the increasing use of MCDM methods in determining optimal materials and processes in the denim industry. Similarly, Majumdar et al. (2010) combined TOPSIS and AHP, two popular MCDM approaches, to select an appropriate navel for rotor-spinning denim fabrics [25].

Feki et al. (2015) proposed a novel approach for physical feature selection based on fuzzy logic and ordered weighted averaging (OWA) operators [26]. Their study on stonew ashed denim provided insights into both objective and subjective evaluations of fabric handles, demonstrating the potential of combining different decision-making techniques. Bai et al. (2021) propose an evaluation framework for buyer–supplier relationship capability from a sustainability perspective and develop a novel visualization method based on DEMATEL and an advanced radar chart to assess and strategize future development in this area, illustrated through a case study in Pakistan’s textile industry [27]. On the other hand, Kaya et al. (2022) integrated DEMATEL and TOPSIS methods as an MCDM approach and Bayesian network for supplier selection [28]. In their study, Sarı et al. (2021) examined factors influencing the purchase of textile products made from recycled materials with the analysis conducted using the DEMATEL method [29].

In addition to the MCDM approach, recent advancements in mathematical modeling for prediction and optimization in denim production have been remarkable. Sarkar et al. (2022) developed a fuzzy logic-based model to predict the tearing strength of laser-engraved denim [30]. Tong et al. (2023) proposed a novel method using convolutional neural networks to predict the parameters of the denim laser fading process [31]. Xu et al. (2020) studied the optimization of production costs for enzyme washing in indigo-dyed cotton denim and demonstrated through the integration of Kriging surrogate and differential evolution algorithms [32]. Xu et al. (2021) presented cost optimization studies for sodium hypochlorite bleaching of indigo-dyed cotton denim, with particle swarm optimization [33]. Gazzah et al. (2015) applied metaheuristic techniques like genetic algorithms and ant colony optimization methods to optimize the behaviors of bagged denim fabric [34]. Ben Fraj and Jaouachi (2022) optimized the bagging behavior of denim garments using the Taguchi approach [35]. Additionally, Katırcıoğlu et al. (2023) predicted quality parameters of denim fabrics using an ANN-based Artificial Bee Colony algorithm hybrid model, illustrating the integration of AI and optimization techniques in enhancing fabric quality [36]. These studies highlight an increasing focus on sustainability, efficiency, and optimization in denim fabric production, utilizing a range of innovative methodologies and analytical approaches. However, there has been limited focus on evaluating denim fabric, especially those incorporating recycled cotton, using a multi-criteria decision-making framework.

In the present study, the use of recycled cotton due to its sustainability benefits constitutes the initial motivation. The integration of recycled cotton into denim fabric production aligns with the industry’s shift towards eco-friendly practices. This multi-criteria decision-making framework evaluates the performance characteristics of denim fabrics containing various levels of recycled cotton, highlighting the balance between sustainability and fabric quality. By addressing the differences between recycled and pristine cotton, this study underlines the importance of sustainable materials in the textile industry and the relevance of the study in promoting eco-friendly practices.

In the view of aforementioned factors, denim fabric structures containing recycled cotton, used for both upper and lower garments, were evaluated as alternatives. Although produced from the same fabric structure, considering the different usage conditions and user expectations for upper and lower garments, criteria expressing the determined structural and performance features of fabric structures for each garment group were separately evaluated based on expert opinions. The criteria weights were obtained using the LMAW method, a new approach that incorporates decision-makers’ opinions and accommodates both qualitative and quantitative data. Additionally, using the determined criteria weights, the ranking of fabric alternatives containing various levels of recycled cotton was conducted through the DNMA method. The results have been generally interpreted through statistical evaluation.

We highlight the study by the following innovative work:

- The study fills critical gaps in denim fabric research, especially concerning the sustainable production and performance evaluation of denim using new multi-criteria decision-making methods.
- Prioritizing the most important performance criteria for denim upper and lower clothes based on expert opinions
- A combined subjective–objective method is used for flexible decision-making.
- The research gap regarding the evaluation of fabric alternatives containing varying ratios of recycled cotton at varying rates is being filled with a multi-criteria decision-making approach.
- The methods employed in the study are recent, and their combined application in prioritizing denim fabric within the textile industry has not been previously implemented.

## 2. Materials and Experimental Design

**Figure 1:**
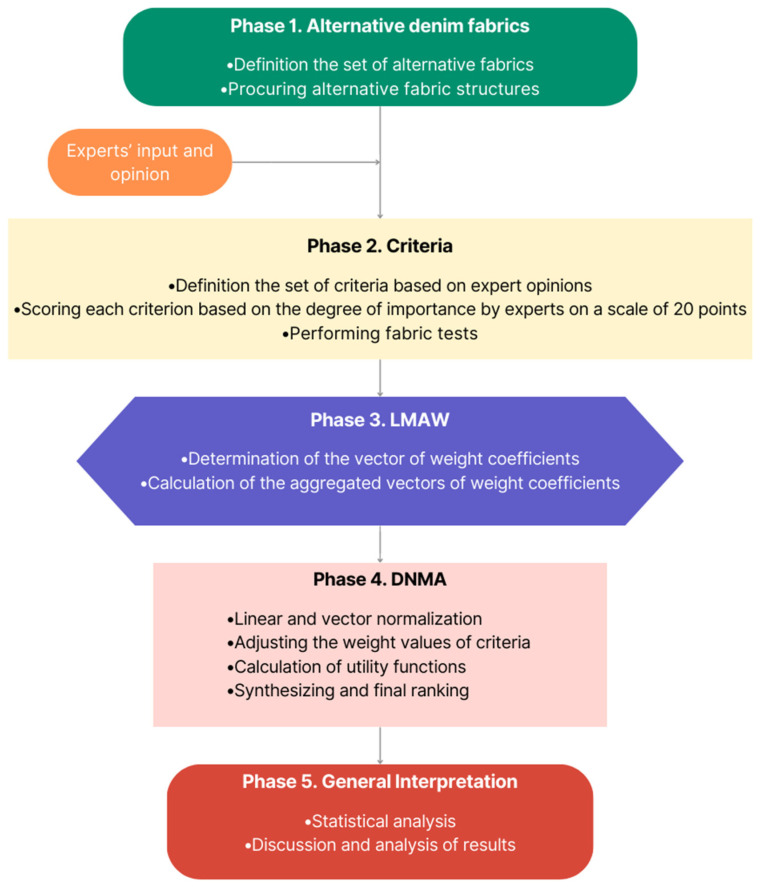
The procedure of the proposed LMAW-DNMA framework.

In the study conducted for the purpose of prioritizing the best denim fabric, the subsequent sections were structured as illustrated in Figure 1 below. Initially, the structural characterization data of fabric alternatives were provided. Afterward, the textile tests to be applied to fabric alternatives and the standards associated with these tests were presented in the Section 3. Following this, the LMAW and DNMA methods used in the study are briefly discussed and explained through calculation steps. The next section is dedicated to the implementation of the work in the context of optimal fabric selection for denim garments. Finally, the statistical analysis and general evaluation of the findings were presented.

### Fabric Alternative Characterization

The alternative denim fabrics produced in a controlled manner were procured from a company operating in Izmir since 1986. Data on the structural properties of the fabric alternatives are presented in Table 1.

In the study, all denim fabric alternatives were subjected to washing under the same conditions. Initially, the fabrics were pre-washed with 1 g/L amylase enzyme, 3 g/L dispersant, and 2.5 g/L anti-crease agent at 50 °C for 12 min. Subsequently, the fabrics underwent a 1 min cold rinse followed by a softening process at 40 °C for 5 min using 5 g/L silicone softener and 2 g/L fixator. Finally, they were dried at 70 °C for 35 min.

## 3. Methodology

### 3.1. Data Collection through Fabric Tests

In the study, various fabric tests were conducted to evaluate the performance characteristics of denim fabric alternatives. The tests, the instruments used, and the standards followed are detailed in Table 2.

The fabric tests provided valuable data on different aspects of the fabric’s performance, which were subsequently used in the multi-criteria decision-making analysis in this study. The mass per unit area tests were performed by cutting into samples of known dimensions and measuring their weights to calculate the mass per unit area. The results were presented in g/m^2^. The fabric thickness was measured using an SDL Atlas Thickness Tester. The fabric samples were placed under a specified pressure, and the thickness was recorded. Results were presented in mm. The kinetic friction coefficient test involved sliding a fabric sample against a reference surface under controlled conditions. The friction coefficient values were recorded and presented as µkin. For tear strength tests, the samples were cut into specific shapes, and force was applied until the fabric tore. The tear strength was measured in N and presented separately for the weft and warp directions. The tensile strength tests were also conducted using the Zwick Z010 machine. Fabric samples were stretched until they broke, and the maximum force was recorded. Results were presented in N for both weft and warp directions. In pilling resistance tests, fabric samples were subjected to controlled rubbing against an abrasive surface, and the formation of pills was evaluated visually. Results were reported using a pilling grade scale from 1 to 5. The abrasion resistance was tested using the same Martindale Tester. The number of cycles, until noticeable wear or damage appeared, was recorded and presented as the number of cycles. Air permeability was measured using the Textest FX 3300 Air Permeability Instrument. The rate of airflow through the fabric under a specified pressure differential was recorded and presented in L/m^2^/s. In rubbing fastness tests, the fabric samples were rubbed against a standard white cloth under both dry and wet conditions. The staining on the white cloth was evaluated and graded from 1 to 5. Dimensional stability tests were conducted using a Wascator. Fabric samples were washed and dried under controlled conditions, and their changes in dimensions were measured. Results were presented as percentage changes in warp and weft directions. In color fastness tests, the fabric samples were subjected to a standard washing procedure, and the degree of color change and staining were assessed visually. Results were reported using a grading scale from 1 to 5.

### 3.2. Integrated MCDM framework for Decision Support

The MCDM methodology for evaluating denim fabric in the study is based on the combination of the LMAW and DNMA methods. The weight of each criterion specific to the relevant product group was determined using the LMAW method. Subsequently, the fabric alternatives were ranked using the DNMA method, employing the criterion weights obtained through LMAW.

#### 3.2.1. LMAW Method Proposed for Ranking the Criteria

The LMAW method, introduced to the academic literature by Pamucar et al. in 2021, represents one of the latest approaches in criterion weighting and alternative ranking, as noted by Demir in 2022 [48,49]. This method is particularly novel, primarily designed for crisp values, as highlighted by Bozanic et al. in 2022 [50]. The LMAW method stands out in dynamic environments for its reliability for decision-makers. One of its most significant contributions is providing maximally consistent and stable results within the MCDM framework, as emphasized by Pamucar et al. in 2021 [48]. In this paper, the focus will be on the aspect of the LMAW method responsible for determining criteria weights. Puska et al. (2023) outline the steps for implementing this method, as follows (Table 3) [51].

$$e:expert;e=1,2,3,\ldots ,k\;j:criterion;j=1,2,3,\ldots ,n\;\eta_{j}^{e}:expert\;opinion\;w_{j}^{e}:weight\;for\;expert\;e\;w_{j}:aggregated\;weight\;p,q:stabilization\;parameters\;of\;Bonferroni\;aggregator;preferably\;1$$

In Table 3, Equation (1) represents the determination of the vector of weight coefficients and this step is performed separately for each expert. The aggregated vectors of weight coefficients are calculated by Equation (2).

#### 3.2.2. DNMA Method Proposed for Ranking the Alternatives

The DNMA, one of the recent methods for ranking alternatives, was introduced to the literature in 2019 by Liao [52]. This technique takes the benefits of two different normalizations (linear and vector) methods and three aggregation functions (Complete Compensation Model—CCM, Non-Compensatory Model—UCM, and Incomplete Compensation Model—ICM) and combines them in an appropriate way [53]. The fundamental principle of the DNMA technique is its ability to bring the preferred alternative closer to the desired solution. This approach is recognized as a significant tool that utilizes the subordinate degree and ranks alternatives using proposed aggregation operators [54,55]. The DNMA method can deal with both quantitative and qualitative criteria, and the benefit, cost, and target-based criteria, simultaneously [52]. It stands out for its credibility, flexibility, and ease of use compared to some recent MCDM methods [54]. Implementation steps of DNMA method are explained briefly in Table 4. DNMA process is in Table 4 [56].

$$i:alternative;i=1,2,3,\ldots ,m\;j:criterion;j=1,2,3,\ldots ,n\;x_{ij}:performance\;value\;x_{ij}^{1}:linear\;normalization\;x_{ij}^{2}:vector\;normalization\;u_{1}\left(a_{i}\right):total\;weighted\;lienar\;normalization\;r_{1}\left(a_{i}\right):first\;type\;rank\;u_{2}\left(a_{i}\right):second\;integration\;function\;for\;linear\;normalization\;r_{2}\left(a_{i}\right):second\;type\;rank\;(ascending\;order)\;u_{3}\left(a_{i}\right):third\;integration\;function\;r_{3}\left(a_{i}\right):third\;type\;rank\;u_{1}^{N}\left(a_{i}\right):first\;normalized\;integration\;function\;u_{2}^{N}\left(a_{i}\right):second\;normalized\;integration\;function\;u_{3}^{N}\left(a_{i}\right):third\;normalized\;integration\;function\;\varphi :importance\;coefficient;preferably\;0.5\;S_{i}:final\;value\;r\left(a_{i}\right):final\;rank$$

### 3.3. General Interpretation through Statistical Analysis

The performance test outcomes derived from each of the fabric alternatives were evaluated using the analysis of variance (ANOVA) tool in SPSS version 20 software, with a 95% confidence interval. Furthermore, the data obtained were also correlated with the results of the DNMA method applied to fabric alternatives and were used in making general evaluations and interpretations.

## 4. Findings

### 4.1. Determination of the Criteria Weights Using LMAW Method

Criterion weight reflects the importance or impact of each criterion in the overall evaluation of the fabric for denim jackets and pants. The orientation and weight of each criterion type play a crucial role in fabric selection and assessment. Criterion weights are assigned based on how critical a particular feature is for the end-use of the garment. On the other hand, criterion orientation type refers to whether a higher or lower value of a specific criterion is desirable or whether they are cost/benefit-oriented. For example, in the case of abrasion resistance, a higher value (indicating greater resistance) is typically preferred, which is a positive orientation. Conversely, for shrinkage, a lower value might be desirable, indicating a negative orientation.

In this study, the textile tests forming the criteria were evaluated by 15 experts separately for jackets and pants production from 1 to 20; where 20 specifies a highly important criterion and 1 specifies the criterion of very low importance. The tables related to the procedures of the LMAW method, used for criterion weighting, are provided in Supplementary Materials (Tables S1–S6). Furthermore, the designation of each criterion as having a high or low value was determined based on expert opinions, and their cost/benefit orientation is presented in Table 5. After the application of the LMAW method, the total weights of all criteria sum up to a value of 1. The criterion weights obtained as a result of applying Equation (2) of the method for denim jackets and pants are presented in Table 5.

Upon examining the criterion weights, it was observed that the weights are generally close in value. However, the differences in the most critical criterion weights reflect the specific requirements for denim jackets and pants. For denim jackets, the most critical criterion identified was color fastness to washing (C15), with a weight of 0.0708. This indicates that maintaining the color quality after washing is highly valued for jackets, which are often exposed to more frequent washing and require a consistent appearance. It was followed by important criteria like dimensional stability in length and width (C13–C14).

In the case of denim pants, the expected properties vary depending on the product group, leading to a different ranking of criterion weights compared to jackets. The most critical criterion identified was abrasion resistance (C9), with a weight of 0.0705. This highlights the importance of durability and resistance to wear for pants, which are subjected to more intense use and friction during everyday activities. These differences in criterion weights demonstrate the tailored approach needed for evaluating denim fabrics for different garment types, considering their unique performance requirements and usage conditions.

### 4.2. Ranking of Alternatives Using DNMA Method

In this research, the primary objective was to identify the fabric with the optimal performance characteristics for the production of denim jackets and pants. The decision matrix (Table 6) used in the DNMA method, which is the same for both garment groups, consists of results from the conducted textile tests. For denim jackets and pants, rankings for each garment group have been separately derived using the criterion orientation and criterion weights provided in Table 5.

The application steps performed separately for denim jackets and denim pants regarding calculated criteria weights, using the above decision matrix, were presented in the Supplementary Materials (Tables S7–S16). Each step of the method involves various mathematical calculations. These steps serve as intermediate stages to reach the final result and are not suitable for interpretation, hence their inclusion in the appendix. The ranking obtained in the final step of the method is used in interpreting the findings.

The final step of the DNMA method involves the calculation of the $S_{i}$ value, which represents the ranking outcomes. The $S_{i}$ value ranges between 0 and 1, with values approaching 1 indicating that an alternative meets the desired performance criteria. Accordingly, based on the $S_{i}$ values, and fabric alternatives have been ranked between 1 and 16. Table 7 shows the $S_{i}$ results; the scores are quite similar for each fabric alternative between the two products, with A5 having the highest score for denim pants and A12 for denim jackets. The top three fabric alternatives indicating that these fabrics meet the desired performance characteristics were ranked A12, A5, and A15 for jacket production, A5, A12, and A15 for pants production, respectively.

**Figure 2:**
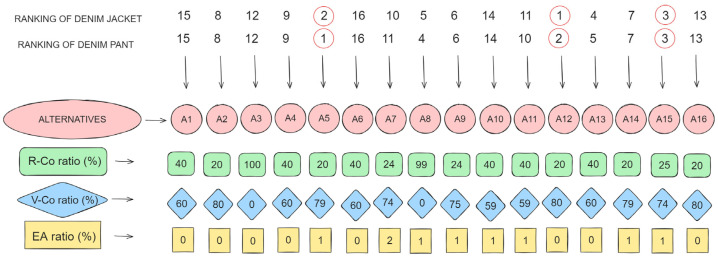
The ranking results of fabric alternatives for both garments regarding fabric content ratio.

To better understand the performance and selection of fabric alternatives for both types of denim products, the ranking results were visualized and presented in Figure 2. The line graph illustrates the ranks for each fabric alternative for both products. The ranks are inversed so that a lower numerical value (which is a higher rank) appears at the top of the graph. There are some variances in rank between the two products for certain fabric alternatives, although the top and bottom-ranked fabric alternatives tend to be similar.

## 5. Discussion

In this section of the research, the fabric alternatives containing recycled cotton, rated optimal for manufacturing denim jackets and pants, were discussed in terms of meeting the desired performance criteria considering the most critical criterion weight.

The aim of multi-criteria decision-making methods is to identify the most suitable alternative that meets several criteria simultaneously, using a comprehensive approach. However, it is not expected that the top-ranked alternatives would meet all the criteria’s cost/benefit orientation simultaneously. Considering the obtained top three ranked fabric alternatives for both garment groups, the findings of this study support this objective.

As highlighted in the literature, tear strength is an important property of the durability of denim products [57,58]. In this study, tear strength is among the examined criteria (C4, C5), and it has been observed that the tear strength values of the top three ranked fabric alternatives (A5, A12, and A15) for both product groups are above the average values of all fabric alternatives. Furthermore, the one-way ANOVA analysis revealed significant variations in the tear strength values in both length (*p* = 0.00) and width (*p* = 0.00) among the fabric alternatives. The *p*-values being less than 0.05 indicate that the variations in tear strength are statistically significant. This suggests that the differences observed in tear strength are unlikely to have occurred by chance, and specific factors such as fabric composition are influencing these variations. Further analysis showed that the presence of elastane in fabric alternatives significantly influences tear strength. Fabrics containing elastane exhibited higher tear strength values compared to those without elastane. This is consistent with the understanding that elastane enhances the flexibility and resilience of the fabric, contributing to greater tear resistance.

The presence of elastane in fabric alternatives, which leads to a higher fractional cover, has been observed to negatively impact air permeability values. This assertion is corroborated by the study conducted by Midha et al. (2017), which revealed that the air permeability of unwashed denim fabrics with cotton weft yarns is significantly higher compared to fabrics with polyester and elastane weft yarns [59]. In the statistical analysis conducted, a significant difference was found in the air permeability values of fabric alternatives when assessed based on elastane content (*p* = 0.000). Similarly, a significant variance was observed in air permeability values when evaluated in relation to R-Co content (*p* = 0.000). These *p*-values indicate that both elastane and recycled cotton content are significant factors affecting air permeability.

In Figure 2, the ranking results obtained from the DNMA method applied to determine the optimal fabrics for the production of denim jackets and pants, along with the content information of each fabric alternative, were visually presented.

As seen in Figure 2, the A8 fabric alternative, containing 99% R-Co, ranked fifth for denim jackets and fourth for denim pants. Similarly, the distribution of rankings for fabric alternatives containing elastane was mixed and did not follow a specific pattern. Therefore, based on the findings of the conducted study, and given that parameters like yarn count and density were not considered stable, it is concluded that the content of recycled cotton and elastane does not significantly impact the ranking when all 15 criteria are evaluated simultaneously.

**Figure 3:**
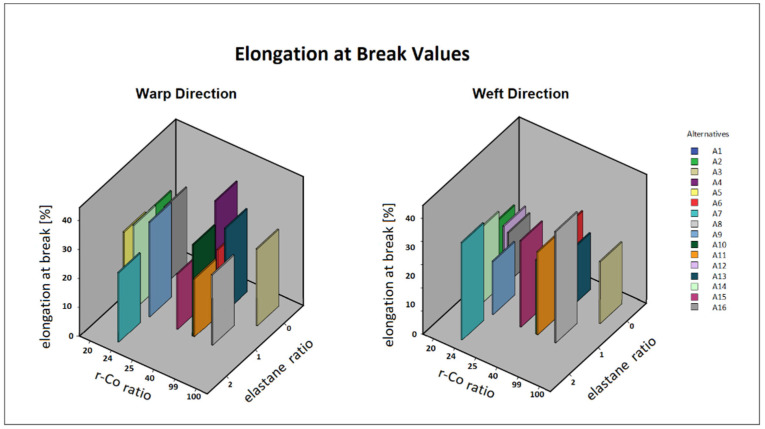
Elongation at break values of fabric alternatives for both warp and weft direction.

Additionally, the elongation at break values, one of the essential properties of denim products, was obtained during the tear strength tests (Figure 3). As illustrated in the graphs, the quantity of elastane incorporated into the denim fabric significantly improves its breaking elongation (*p* = 0.00), similar to findings reported in the literature [60,61]. Typically, elastane is fed into the weft yarns in woven fabrics. Consequently, as expected, fabrics containing elastane have shown higher values in the weft direction for elongation at break. However, the elongation at break in the warp direction does not exhibit a similar correlation with the elastane content and is thought to be more related to the yarn properties. From these findings, it can be inferred that elastane enhances fabric stretchability and comfort properties, while the use of recycled materials may impact the mechanical strength and elongation characteristics of the fabric.

## 6. Conclusions

The current study provides significant insights into the sustainable production and performance evaluation of denim using novel MCDM methods. It employs a combined MCDM approach merging the LMAW method for criterion weight determination and the DNMA method for optimal fabric selection in jacket and pant production. In the study, it was emphasized the importance of various criteria, such as tear strength, tensile strength, air permeability, color fastness, abrasion resistance, etc., reflecting the unique demands of denim jackets and pants. These factors are assessed collectively to ascertain the fabric’s overall performance and suitability for the intended garment type. It is obtained that a higher weight on color fastness to washing (C15) for denim jackets is an attribute that is highly valued for jacket fabrics, while abrasion resistance (C9) was more prioritized for pants, reflecting their respective usage requirements and durability expectations.

The DNMA method’s application in ranking alternatives brings a new dimension to the evaluation. Considering ranking results, the close ranking scores among different fabrics for both products indicate a comprehensive performance alignment with the set criteria. The top three fabric alternatives for denim production were identified as A12, A5, and A15, respectively. Similarly, for pants production, the best-performing fabrics were identified as A5, A12, and A15, respectively. This ranking highlights the fabrics that most closely meet the desired performance attributes for each type of garment. The results of this research support the aim of MCDM methods identifying the most suitable materials while considering multiple criteria, although no single alternative meets all criteria perfectly. The top-ranked denim fabric alternatives align well with the study’s criterion orientations, demonstrating the practical application of these methods in the textile industry.

This study demonstrates the importance of sustainable practices in the denim industry, especially through the use of recycled cotton. Therefore, the selected alternative fabrics, conducted textile tests, applied methods, and expert evaluations constitute the limitations of this study. As the applied models have their own process steps, different results can be obtained with different models. This expresses the uniqueness of each method. Additionally, over the years, as the shortcomings of the methods used have been identified and improved upon, new methods have been developed, leading to more reliable results.

Consequently, the field of sustainability has great potential for the application of the MCDM approach. Therefore, future studies could explore further sustainable materials and innovative production techniques.

## Figures and Tables

**Table 1:** Fabric characteristics.

Fabric Alternatives	Fabric Composition	Yarn Count		Density [yarns/cm]		Weave Type
		Weft	Warp	Weft	Warp	
A1	40% R-Co, 60% V-Co	Ne 12/1	12/1 Ne	17	28	3/1 Twill (Z)
A2	20% R-Co, 80% V-Co	Ne 12/1	12/1 Ne	16	24	
A3	100% R-Co	Ne 12/1	8/1 Ne	18	28	
A4	40% R-Co, 60% V-Co	Ne 12/1	10/1 Ne	17	23	
A5	20% R-Co, 79% V-Co, 1% Ea	Ne 12/1 + 40 dtex Ea	10/1 Ne	18	27	
A6	40% R-Co, 60% V-Co	Ne 12/1	10/1 Ne	18	23	
A7	24% R-Co, 74% V-Co, %2 Ea	Ne 12/1 + 72 dtex Ea	10/1 Ne	19	27	
A8	99% R-Co, 1% Ea	Ne 12/1 + 78 dtex Ea	12/1 Ne	18	25	
A9	24% R-Co, 75% V-Co, 1% Ea	Ne 12/1 + 72 dtex Ea	10/1 Ne	19	27	
A10	40% R-Co, 59% V-Co, 1% Ea	Ne 8/1 + 78 dtex Ea	10/1 Ne	19	26	
A11	40% R-Co, 59% V-Co, 1% Ea	Ne 12/1 + 72 dtex Ea	9/1 Ne	18	25	
A12	20% R-Co, 80% V-Co	Ne 12/1	12/1 Ne	18	23	
A13	40% R-Co, 60% V-Co	Ne 12/1	8/1 Ne	20	28	
A14	20% R-Co, 79% V-Co, 1% Ea	Ne 12/1 + 78 dtex Ea	9/1 Ne	17	25	
A15	25% R-Co, 74% V-Co, 1% Ea	Ne 14/1 + 40 dtex Ea	12/1 Ne	21	28	
A16	20% R-Co, 80% V-Co	Ne 12/1	10/1 Ne	17	23	
R-Co: Recycled Cotton; V-Co: Virgin Cotton; Ea: Elastane						

**Table 2:** The fabric tests for data collection.

Fabric Tests	Testing Instrument	Manufacturer/Developer	Country	Test Standard	Year of Issue
Mass per unit area	Sartorius Scales	Sartorius	Germany	EN 12127 [37]	1997
Fabric thickness	SDL Atlas	SDL Atlas	USA	ISO 5084 [38]	1996
Kinetic friction coefficient	Frictorq (Fabric Friction Tester)	University of Minho	Portugal	ISO 21182 [39]	2013
Tear strength	Zwick Z010 (Roell) tensile strength testing machine	Zwick Roell	Germany	EN ISO 13937-2 [40]	2000
Tensile strength	Zwick Z010 (Roell) tensile strength testing machine	Zwick Roell	Germany	EN ISO 13934-1 [41]	2013
Pilling resistance	Martindale Pilling and Abrasion Tester	James H. Heal	UK	EN ISO 12945-2 [42]	2020
Abrasion resistance	Martindale Pilling and Abrasion Tester	James H. Heal	UK	EN ISO 7784-2 [43]	2023
Air permeability	Textest FX 3300 air permeability instrument	Textest	Switzerland	ISO 9237 [44]	1995
Rubbing fastness (dry and wet)	Crockmeter	SDL Atlas	USA	TS EN ISO 105-X12 [45]	2016
Dimensional stability	Wascator	Electrolux	Sweden	EN ISO 3759 [46]	2011
Color fastness to washing	Atlas Linitest Plus	SDL Atlas	USA	EN ISO 105-C06 [47]	2010

**Table 3:** LMAW process [37].

Step	Equation	Equation Number
Weight for expert e	$w_{j}^{e}=\frac{\ln\left[\eta_{j}^{e}\right]}{\ln\left[\prod_{j=1}^{n}\eta_{j}^{e}\right]}$	(1)
Aggregated weight	$w_{j}={\left[\frac{1}{k\left(k-1\right)}\sum_{x=1}^{k}{\left(w_{j}^{x}\right)}^{p}\sum_{y=1;y\neq x}^{k}{\left(w_{j}^{y}\right)}^{q}\right]}^{\left(\frac{1}{p+q}\right)}$	(2)

**Table 4:** DNMA process.

Step	Equation	Equation No
Linear normalization (benefit criterion)	$x_{ij}^{1}=1-\left|\frac{x_{ij}-\underset{j}{\max}x_{ij}}{\underset{j}{\max}x_{ij}-\underset{j}{\min}x_{ij}}\right|$	(3)
Linear normalization (cost criterion)	$x_{ij}^{1}=1-\left|\frac{x_{ij}-\underset{j}{\min}x_{ij}}{\underset{j}{\max}x_{ij}-\underset{j}{\min}x_{ij}}\right|$	(4)
Vector normalization (benefit criterion)	$x_{ij}^{2}=1-\frac{\left|x_{ij}-\underset{j}{\max}x_{ij}\right|}{\sqrt{\sum_{i=1}^{m}{\left(x_{ij}\right)}^{2}+{\left(\underset{j}{\max}x_{ij}\right)}^{2}}}$	(5)
Vector normalization (cost criterion)	$x_{ij}^{2}=1-\frac{\left|x_{ij}-\underset{j}{\min}x_{ij}\right|}{\sqrt{\sum_{i=1}^{m}{\left(x_{ij}\right)}^{2}+{\left(\underset{j}{\min}x_{ij}\right)}^{2}}}$	(6)
Total weighted linear normalization	$u_{1}\left(a_{i}\right)=\sum_{j=1}^{n}\left[w_{j}x_{ij}^{1}\right]$	(7)
Second integration function for linear normalization	$u_{2}\left(a_{i}\right)=\underset{j}{max}\left[w_{j}\left(1-x_{ij}^{1}\right)\right]$	(8)
Second integration function (vector normalization)	$u_{3}\left(a_{i}\right)=\prod_{j=1}^{n}\left[{x_{ij}^{2}}^{w_{j}}\right]$	(9)
The first normalized integration function	$u_{1}^{N}\left(a_{i}\right)=\frac{u_{1}\left(a_{i}\right)}{\sqrt{\sum_{i=1}^{m}{\left[u_{1}\left(a_{i}\right)\right]}^{2}}}$	(10)
The second normalized integration function	$u_{2}^{N}\left(a_{i}\right)=\frac{u_{2}\left(a_{i}\right)}{\sqrt{\sum_{i=1}^{m}{\left[u_{2}\left(a_{i}\right)\right]}^{2}}}$	(11)
The third normalized integration function	$u_{3}^{N}\left(a_{i}\right)=\frac{u_{3}\left(a_{i}\right)}{\sqrt{\sum_{i=1}^{m}{\left[u_{3}\left(a_{i}\right)\right]}^{2}}}$	(12)
Final value	$\begin{array}{c} S_{i}=\sqrt{\varphi {\left[u_{1}^{N}\left(a_{i}\right)\right]}^{2}+(1-\varphi ){\left[\frac{m-r_{1}\left(a_{i}\right)+1}{\frac{m(m+1)}{2}}\right]}^{2}}\\ \;\;\;-\sqrt{\varphi {\left[u_{2}^{N}\left(a_{i}\right)\right]}^{2}+\left(1-\varphi \right){\left[\frac{r_{2}\left(a_{i}\right)}{\frac{m\left(m+1\right)}{2}}\right]}^{2}}\\ \;\;\;+\sqrt{\varphi {\left[u_{3}^{N}\left(a_{i}\right)\right]}^{2}+(1-\varphi ){\left[\frac{m-r_{3}\left(a_{i}\right)+1}{\frac{m(m+1)}{2}}\right]}^{2}} \end{array}$	(13)

**Table 5:** Criterion type orientation and criterion weight of each criterion regarding denim jackets and denim pants.

Criterion No	Criterion	Criterion Orientation Type		Criterion Weight ($w_{j}$)	
		Denim Jacket	Denim Pant	Denim Jacket	Denim Pant
C1	Mass per unit area	min.	min.	0.0635	0.0620
C2	Fabric thickness	min.	min.	0.0622	0.0620
C3	Kinetic friction coefficient	min.	min.	0.0633	0.0642
C4	Tear strength/weft wise	max.	max.	0.0672	0.0680
C5	Tear strength/warp wise	max.	max.	0.0667	0.0675
C6	Tensile strength/weft wise	max.	max.	0.0663	0.0676
C7	Tensile strength/warp wise	max.	max.	0.0662	0.0674
C8	Pilling resistance	max.	max.	0.0660	0.0642
C9	Abrasion resistance	max.	max.	0.0685	0.0705
C10	Air permeability	max.	max.	0.0661	0.0632
C11	Dry rubbing fastness	max.	max.	0.0672	0.0681
C12	Wet rubbing fastness	max.	max.	0.0665	0.0678
C13	Dimensional stability/weft wise	min.	min.	0.0698	0.0685
C14	Dimensional stability/warp wise	min.	min.	0.0697	0.0687
C15	Color fastness to washing	max.	max.	0.0708	0.0699

**Table 6:** Decision matrix for sustainable denim fabric alternatives.

Alt. No.	Criteria														
	C1 (g/m^2^)	C2 (mm)	C3 (µkin)	C4 (N)	C5 (N)	C6 (N)	C7 (N)	C8 (Grade)	C9 (Cycle)	C10 (L/m^2^/s)	C11 (Grade)	C12 (Grade)	C13 (%)	C14 (%)	C15 (Grade)
A1	403.88	0.866	0.2837	32.67	39.43	390.64	1040.99	3.5	203.33	86.65	4.5	1.50	3.0	2.0	4.5
A2	417.40	0.828	0.2884	39.67	46.17	530.68	1404.01	4.5	146.67	54.97	4.0	1.00	0.5	1.0	4.5
A3	419.32	0.814	0.2849	39.63	53.30	600.98	1429.52	4.5	120.00	30.56	4.0	1.00	1.0	0.5	4.5
A4	470.84	0.952	0.2800	34.20	48.70	523.38	1346.03	3.5	483.33	82.70	4.0	1.00	0.5	2.0	4.5
A5	413.20	0.797	0.2576	54.37	51.67	531.70	1512.05	3.5	143.33	44.34	4.5	2.00	3.5	2.0	5.0
A6	369.04	0.726	0.2781	31.60	45.43	514.93	1162.21	3.5	126.67	65.66	2.5	1.00	0.	4.0	4.5
A7	347.76	0.742	0.2767	37.03	48.47	469.42	1250.45	4.5	133.33	58.53	4.5	2.50	2.0	9.5	4.5
A8	439.26	0.837	0.2586	56.60	45.97	562.34	1288.56	4.5	143.33	35.14	4.5	2.00	0.0	4.0	4.5
A9	399.36	0.802	0.2935	37.93	150.00	480.33	1430.27	4.5	133.33	38.17	4.5	2.00	2.0	6.5	4.5
A10	474.42	0.892	0.2697	47.20	62.13	586.31	1912.35	3.5	293.33	37.12	4.5	1.00	4.0	2.0	4.5
A11	413.78	0.733	0.3057	50.43	66.73	748.02	1516.19	3.5	220.00	22.02	3.5	1.00	0.0	0.0	4.5
A12	372.02	0.741	0.2664	65.87	83.17	772.53	1388.54	3.5	53.33	55.62	4.5	2.50	1.5	3.0	4.5
A13	367.26	0.760	0.2565	19.27	150.00	293.82	1268.75	4.5	146.67	53.50	3.5	1.00	0.5	0.75	4.5
A14	455.22	0.788	0.2682	53.57	69.00	917.03	1964.87	4.5	236.67	24.06	4.0	2.00	6.0	6.0	4.5
A15	323.60	0.625	0.2715	30.13	150.00	358.76	1486.55	3.5	83.33	38.67	4.5	3.00	1.75	5.0	4.5
A16	420.82	0.866	0.2804	44.07	42.03	499.69	1247.41	4.5	60.00	80.43	3.0	1.00	1.0	0.0	4.5

**Table 7:** Ranking results of fabric alternatives for denim jacket and denim pant production.

Fabric Alternatives	Denim Jacket		Denim Pant	
	$S_{i}$	Rank	$S_{i}$	Rank
A1	0.1339	15	0.1337	15
A2	0.1802	8	0.1797	8
A3	0.1703	12	0.1704	12
A4	0.1754	9	0.1754	9
A5	0.2289	2	0.2321	1
A6	0.1233	16	0.1226	16
A7	0.1744	10	0.1746	11
A8	0.2114	5	0.2122	4
A9	0.1867	6	0.1876	6
A10	0.1495	14	0.1523	14
A11	0.1740	11	0.1750	10
A12	0.2405	1	0.2273	2
A13	0.2116	4	0.2111	5
A14	0.1836	7	0.1862	7
A15	0.2192	3	0.2205	3
A16	0.1614	13	0.1599	13

## Data Availability

Data are contained within the article.

## Supplementary Materials

The following supporting information can be downloaded at: https://www.mdpi.com/article/10.3390/ma17133291/s1, Table S1: Initial matrix obtained expert scores for denim jacket; Table S2 Weights for each expert for denim jacket using Equation (1); Table S3: Aggregated weights for denim jacket using Equation (2); Table S4: Initial matrix obtained expert scores for denim pant; Table S5: Weights for each expert for denim pant using Equation (1); Table S6: Aggregated weights for denim pant using Equation (2); Table S7: Linear normalization (cost & benefit criterion) for denim jacket using Equations (3) and (4); Table S8: Vector normalization (cost & benefit criterion) for denim jacket using Equations (5) and (6); Table S9: Calculation of $w_{j}\left(1-x_{ij}^{1}\right)$ for denim jacket; Table S10: Calculation of $\left[{x_{ij}^{2}}^{w_{j}}\right]$ for denim jacket; Table S11: Ranking results obtained using Equations (7)–(13) for denim jacket; Table S12: Linear normalization (cost & benefit criterion) for denim pant using Equations (3) and (4); Table S13: Vector normalization (cost & benefit criterion) for denim pant using Equations (5) and (6); Table S14: Calculation of $w_{j}\left(1-x_{ij}^{1}\right)$ for denim pant; Table S15: Calculation of $\left[{x_{ij}^{2}}^{w_{j}}\right]$ for denim pant; Table S16: Ranking results obtained using Equations (7)–(13) for denim pant.
