# Supplementary material for: Prioritizing Sustainable Denim Fabric through Integrated Decision-Making Framework
Source: Materials (Basel). 2024 Jul 3;17(13):3291. doi: 10.3390/ma17133291 (PMC11243534; doi:10.3390/ma17133291)
Supplement: Supplementary file 1 [file materials-17-03291-s001.zip › materials-2998275-supplementary.pdf]

Supplementary materials

Table S1 Initial matrix obtained expert scores for denim jacket

	C1	C2	C3	C4	C5	C6	C7	C8	C9	C10	C11	C12	C13	C14	C15
**E1**	10	8	15	15	15	15	15	16	18	15	15	15	18	18	20
**E2**	12	13	9	10	10	8	8	11	17	20	12	13	16	16	15
**E3**	20	20	14	13	13	17	17	11	15	14	10	11	9	10	12
**E4**	10	9	14	20	20	20	20	13	15	16	17	15	18	18	14
**E5**	20	18	15	15	15	12	12	12	12	16	15	12	18	17	20
**E6**	17	15	16	15	15	14	14	15	15	13	14	13	13	13	18
**E7**	15	15	10	20	20	20	20	15	15	10	15	15	20	20	20
**E8**	15	10	10	13	13	10	10	18	10	15	15	16	16	15	18
**E9**	15	15	10	20	20	20	20	15	17	15	15	15	20	20	20
**E10**	10	15	13	16	15	15	16	18	15	5	12	9	18	18	18
**E11**	12	12	17	13	13	11	11	7	19	19	18	18	18	18	19
**E12**	10	9	18	11	10	16	15	19	20	19	20	20	18	19	19
**E13**	11	9	8	20	20	20	20	19	18	20	19	20	19	18	18
**E14**	11	11	18	19	18	16	17	18	20	15	20	20	19	18	17
**E15**	15	15	15	16	14	16	14	20	20	18	17	17	20	20	20

Table S2 Weights for each expert for denim jacket using Equation 1

	C1	C2	C3	C4	C5	C6	C7	C8	C9	C10	C11	C12	C13	C14	C15
**1**	0.0569	0.0514	0.0669	0.0669	0.0669	0.0669	0.0669	0.0685	0.0714	0.0669	0.0669	0.0669	0.0714	0.0714	0.0740
**2**	0.0662	0.0683	0.0585	0.0613	0.0613	0.0554	0.0554	0.0639	0.0755	0.0798	0.0662	0.0683	0.0739	0.0739	0.0721
**3**	0.0771	0.0771	0.0679	0.0660	0.0660	0.0729	0.0729	0.0617	0.0697	0.0679	0.0593	0.0617	0.0565	0.0593	0.0639
**4**	0.0560	0.0534	0.0642	0.0728	0.0728	0.0728	0.0728	0.0624	0.0658	0.0674	0.0689	0.0658	0.0703	0.0703	0.0642
**5**	0.0737	0.0711	0.0666	0.0666	0.0666	0.0612	0.0612	0.0612	0.0612	0.0682	0.0666	0.0612	0.0711	0.0697	0.0737
**6**	0.0705	0.0673	0.0689	0.0673	0.0673	0.0656	0.0656	0.0673	0.0673	0.0638	0.0656	0.0638	0.0638	0.0638	0.0719
**7**	0.0647	0.0647	0.0551	0.0716	0.0716	0.0716	0.0716	0.0647	0.0647	0.0551	0.0647	0.0647	0.0716	0.0716	0.0716
**8**	0.0698	0.0593	0.0593	0.0661	0.0661	0.0593	0.0593	0.0745	0.0593	0.0698	0.0698	0.0715	0.0715	0.0698	0.0745
**9**	0.0639	0.0639	0.0544	0.0707	0.0707	0.0707	0.0707	0.0639	0.0669	0.0639	0.0639	0.0639	0.0707	0.0707	0.0707

**10**	0.0589	0.0693	0.0656	0.0709	0.0693	0.0693	0.0709	0.0739	0.0693	0.0412	0.0636	0.0562	0.0739	0.0739	0.0739
**11**	0.0620	0.0620	0.0707	0.0640	0.0640	0.0598	0.0598	0.0486	0.0735	0.0735	0.0721	0.0721	0.0721	0.0721	0.0735
**12**	0.0559	0.0533	0.0701	0.0582	0.0559	0.0673	0.0657	0.0714	0.0727	0.0714	0.0727	0.0727	0.0701	0.0714	0.0714
**13**	0.0569	0.0521	0.0493	0.0711	0.0711	0.0711	0.0711	0.0699	0.0686	0.0711	0.0699	0.0711	0.0699	0.0686	0.0686
**14**	0.0566	0.0566	0.0682	0.0695	0.0682	0.0654	0.0669	0.0682	0.0707	0.0639	0.0707	0.0707	0.0695	0.0682	0.0669
**15**	0.0637	0.0637	0.0637	0.0653	0.0621	0.0653	0.0621	0.0705	0.0705	0.0680	0.0667	0.0667	0.0705	0.0705	0.0705

Table S3 Aggregated weights for denim jacket using Equation 2

**E**	**E**	**C1**	**C2**	**C3**	**C4**	**C5**	**C6**	**C7**	**C8**	**C9**	**C10**	**C11**	**C12**	**C13**	**C14**	**C15**
**1**	2	0.0038	0.0035	0.0039	0.0041	0.0041	0.0037	0.0037	0.0044	0.0054	0.0053	0.0044	0.0046	0.0053	0.0053	0.0053
**1**	3	0.0044	0.0040	0.0045	0.0044	0.0044	0.0049	0.0049	0.0042	0.0050	0.0045	0.0040	0.0041	0.0040	0.0042	0.0047
**1**	4	0.0032	0.0027	0.0043	0.0049	0.0049	0.0049	0.0049	0.0043	0.0047	0.0045	0.0046	0.0044	0.0050	0.0050	0.0047
**1**	5	0.0042	0.0037	0.0045	0.0045	0.0045	0.0041	0.0041	0.0042	0.0044	0.0046	0.0045	0.0041	0.0051	0.0050	0.0055
**1**	6	0.0040	0.0035	0.0046	0.0045	0.0045	0.0044	0.0044	0.0046	0.0048	0.0043	0.0044	0.0043	0.0046	0.0046	0.0053
**1**	7	0.0037	0.0033	0.0037	0.0048	0.0048	0.0048	0.0048	0.0044	0.0046	0.0037	0.0043	0.0043	0.0051	0.0051	0.0053
**1**	8	0.0040	0.0030	0.0040	0.0044	0.0044	0.0040	0.0040	0.0051	0.0042	0.0047	0.0047	0.0048	0.0051	0.0050	0.0055
**1**	9	0.0036	0.0033	0.0036	0.0047	0.0047	0.0047	0.0047	0.0044	0.0048	0.0043	0.0043	0.0043	0.0050	0.0050	0.0052
**1**	10	0.0033	0.0036	0.0044	0.0047	0.0046	0.0046	0.0047	0.0051	0.0049	0.0028	0.0043	0.0038	0.0053	0.0053	0.0055
**1**	11	0.0035	0.0032	0.0047	0.0043	0.0043	0.0040	0.0040	0.0033	0.0052	0.0049	0.0048	0.0048	0.0051	0.0051	0.0054
**1**	12	0.0032	0.0027	0.0047	0.0039	0.0037	0.0045	0.0044	0.0049	0.0052	0.0048	0.0049	0.0049	0.0050	0.0051	0.0053
**1**	13	0.0032	0.0027	0.0033	0.0048	0.0048	0.0048	0.0048	0.0048	0.0049	0.0048	0.0047	0.0048	0.0050	0.0049	0.0051
**1**	14	0.0032	0.0029	0.0046	0.0046	0.0046	0.0044	0.0045	0.0047	0.0050	0.0043	0.0047	0.0047	0.0050	0.0049	0.0049
**1**	15	0.0036	0.0033	0.0043	0.0044	0.0042	0.0044	0.0042	0.0048	0.0050	0.0046	0.0045	0.0045	0.0050	0.0050	0.0052
**2**	1	0.0038	0.0035	0.0039	0.0041	0.0041	0.0037	0.0037	0.0044	0.0054	0.0053	0.0044	0.0046	0.0053	0.0053	0.0053
**2**	3	0.0051	0.0053	0.0040	0.0040	0.0040	0.0040	0.0040	0.0039	0.0053	0.0054	0.0039	0.0042	0.0042	0.0044	0.0046
**2**	4	0.0037	0.0037	0.0038	0.0045	0.0045	0.0040	0.0040	0.0040	0.0050	0.0054	0.0046	0.0045	0.0052	0.0052	0.0046
**2**	5	0.0049	0.0049	0.0039	0.0041	0.0041	0.0034	0.0034	0.0039	0.0046	0.0054	0.0044	0.0042	0.0053	0.0051	0.0053
**2**	6	0.0047	0.0046	0.0040	0.0041	0.0041	0.0036	0.0036	0.0043	0.0051	0.0051	0.0043	0.0044	0.0047	0.0047	0.0052
**2**	7	0.0043	0.0044	0.0032	0.0044	0.0044	0.0040	0.0040	0.0041	0.0049	0.0044	0.0043	0.0044	0.0053	0.0053	0.0052
**2**	8	0.0046	0.0041	0.0035	0.0041	0.0041	0.0033	0.0033	0.0048	0.0045	0.0056	0.0046	0.0049	0.0053	0.0052	0.0054

**2**	9	0.0042	0.0044	0.0032	0.0043	0.0043	0.0039	0.0039	0.0041	0.0050	0.0051	0.0042	0.0044	0.0052	0.0052	0.0051
**2**	10	0.0039	0.0047	0.0038	0.0043	0.0042	0.0038	0.0039	0.0047	0.0052	0.0033	0.0042	0.0038	0.0055	0.0055	0.0053
**2**	11	0.0041	0.0042	0.0041	0.0039	0.0039	0.0033	0.0033	0.0031	0.0055	0.0059	0.0048	0.0049	0.0053	0.0053	0.0053
**2**	12	0.0037	0.0036	0.0041	0.0036	0.0034	0.0037	0.0036	0.0046	0.0055	0.0057	0.0048	0.0050	0.0052	0.0053	0.0052
**2**	13	0.0038	0.0036	0.0029	0.0044	0.0044	0.0039	0.0039	0.0045	0.0052	0.0057	0.0046	0.0049	0.0052	0.0051	0.0049
**2**	14	0.0037	0.0039	0.0040	0.0043	0.0042	0.0036	0.0037	0.0044	0.0053	0.0051	0.0047	0.0048	0.0051	0.0050	0.0048
**2**	15	0.0042	0.0044	0.0037	0.0040	0.0038	0.0036	0.0034	0.0045	0.0053	0.0054	0.0044	0.0046	0.0052	0.0052	0.0051
**3**	1	0.0044	0.0040	0.0045	0.0044	0.0044	0.0049	0.0049	0.0042	0.0050	0.0045	0.0040	0.0041	0.0040	0.0042	0.0047
**3**	2	0.0051	0.0053	0.0040	0.0040	0.0040	0.0040	0.0040	0.0039	0.0053	0.0054	0.0039	0.0042	0.0042	0.0044	0.0046
**3**	4	0.0043	0.0041	0.0044	0.0048	0.0048	0.0053	0.0053	0.0038	0.0046	0.0046	0.0041	0.0041	0.0040	0.0042	0.0041
**3**	5	0.0057	0.0055	0.0045	0.0044	0.0044	0.0045	0.0045	0.0038	0.0043	0.0046	0.0039	0.0038	0.0040	0.0041	0.0047
**3**	6	0.0054	0.0052	0.0047	0.0044	0.0044	0.0048	0.0048	0.0042	0.0047	0.0043	0.0039	0.0039	0.0036	0.0038	0.0046
**3**	7	0.0050	0.0050	0.0037	0.0047	0.0047	0.0052	0.0052	0.0040	0.0045	0.0037	0.0038	0.0040	0.0041	0.0042	0.0046
**3**	8	0.0054	0.0046	0.0040	0.0044	0.0044	0.0043	0.0043	0.0046	0.0041	0.0047	0.0041	0.0044	0.0040	0.0041	0.0048
**3**	9	0.0049	0.0049	0.0037	0.0047	0.0047	0.0052	0.0052	0.0039	0.0047	0.0043	0.0038	0.0039	0.0040	0.0042	0.0045
**3**	10	0.0045	0.0053	0.0045	0.0047	0.0046	0.0051	0.0052	0.0046	0.0048	0.0028	0.0038	0.0035	0.0042	0.0044	0.0047
**3**	11	0.0048	0.0048	0.0048	0.0042	0.0042	0.0044	0.0044	0.0030	0.0051	0.0050	0.0043	0.0045	0.0041	0.0043	0.0047
**3**	12	0.0043	0.0041	0.0048	0.0038	0.0037	0.0049	0.0048	0.0044	0.0051	0.0049	0.0043	0.0045	0.0040	0.0042	0.0046
**3**	13	0.0044	0.0040	0.0034	0.0047	0.0047	0.0052	0.0052	0.0043	0.0048	0.0048	0.0041	0.0044	0.0039	0.0041	0.0044
**3**	14	0.0044	0.0044	0.0046	0.0046	0.0045	0.0048	0.0049	0.0042	0.0049	0.0043	0.0042	0.0044	0.0039	0.0040	0.0043
**3**	15	0.0049	0.0049	0.0043	0.0043	0.0041	0.0048	0.0045	0.0044	0.0049	0.0046	0.0040	0.0041	0.0040	0.0042	0.0045
**4**	1	0.0032	0.0027	0.0043	0.0049	0.0049	0.0049	0.0049	0.0043	0.0047	0.0045	0.0046	0.0044	0.0050	0.0050	0.0047
**4**	2	0.0037	0.0037	0.0038	0.0045	0.0045	0.0040	0.0040	0.0040	0.0050	0.0054	0.0046	0.0045	0.0052	0.0052	0.0046
**4**	3	0.0043	0.0041	0.0044	0.0048	0.0048	0.0053	0.0053	0.0038	0.0046	0.0046	0.0041	0.0041	0.0040	0.0042	0.0041
**4**	5	0.0041	0.0038	0.0043	0.0049	0.0049	0.0045	0.0045	0.0038	0.0040	0.0046	0.0046	0.0040	0.0050	0.0049	0.0047
**4**	6	0.0039	0.0036	0.0044	0.0049	0.0049	0.0048	0.0048	0.0042	0.0044	0.0043	0.0045	0.0042	0.0045	0.0045	0.0046
**4**	7	0.0036	0.0035	0.0035	0.0052	0.0052	0.0052	0.0052	0.0040	0.0043	0.0037	0.0045	0.0043	0.0050	0.0050	0.0046
**4**	8	0.0039	0.0032	0.0038	0.0048	0.0048	0.0043	0.0043	0.0046	0.0039	0.0047	0.0048	0.0047	0.0050	0.0049	0.0048
**4**	9	0.0036	0.0034	0.0035	0.0052	0.0052	0.0052	0.0052	0.0040	0.0044	0.0043	0.0044	0.0042	0.0050	0.0050	0.0045
**4**	10	0.0033	0.0037	0.0042	0.0052	0.0050	0.0050	0.0052	0.0046	0.0046	0.0028	0.0044	0.0037	0.0052	0.0052	0.0047

**4**	11	0.0035	0.0033	0.0045	0.0047	0.0047	0.0044	0.0044	0.0030	0.0048	0.0050	0.0050	0.0047	0.0051	0.0051	0.0047
**4**	12	0.0031	0.0028	0.0045	0.0042	0.0041	0.0049	0.0048	0.0045	0.0048	0.0048	0.0050	0.0048	0.0049	0.0050	0.0046
**4**	13	0.0032	0.0028	0.0032	0.0052	0.0052	0.0052	0.0052	0.0044	0.0045	0.0048	0.0048	0.0047	0.0049	0.0048	0.0044
**4**	14	0.0032	0.0030	0.0044	0.0051	0.0050	0.0048	0.0049	0.0043	0.0047	0.0043	0.0049	0.0047	0.0049	0.0048	0.0043
**4**	15	0.0036	0.0034	0.0041	0.0048	0.0045	0.0048	0.0045	0.0044	0.0046	0.0046	0.0046	0.0044	0.0050	0.0050	0.0045
**5**	1	0.0042	0.0037	0.0045	0.0045	0.0045	0.0041	0.0041	0.0042	0.0044	0.0046	0.0045	0.0041	0.0051	0.0050	0.0055
**5**	2	0.0049	0.0049	0.0039	0.0041	0.0041	0.0034	0.0034	0.0039	0.0046	0.0054	0.0044	0.0042	0.0053	0.0051	0.0053
**5**	3	0.0057	0.0055	0.0045	0.0044	0.0044	0.0045	0.0045	0.0038	0.0043	0.0046	0.0039	0.0038	0.0040	0.0041	0.0047
**5**	4	0.0041	0.0038	0.0043	0.0049	0.0049	0.0045	0.0045	0.0038	0.0040	0.0046	0.0046	0.0040	0.0050	0.0049	0.0047
**5**	6	0.0052	0.0048	0.0046	0.0045	0.0045	0.0040	0.0040	0.0041	0.0041	0.0044	0.0044	0.0039	0.0045	0.0044	0.0053
**5**	7	0.0048	0.0046	0.0037	0.0048	0.0048	0.0044	0.0044	0.0040	0.0040	0.0038	0.0043	0.0040	0.0051	0.0050	0.0053
**5**	8	0.0051	0.0042	0.0040	0.0044	0.0044	0.0036	0.0036	0.0046	0.0036	0.0048	0.0047	0.0044	0.0051	0.0049	0.0055
**5**	9	0.0047	0.0045	0.0036	0.0047	0.0047	0.0043	0.0043	0.0039	0.0041	0.0044	0.0043	0.0039	0.0050	0.0049	0.0052
**5**	10	0.0043	0.0049	0.0044	0.0047	0.0046	0.0042	0.0043	0.0045	0.0042	0.0028	0.0042	0.0034	0.0053	0.0052	0.0055
**5**	11	0.0046	0.0044	0.0047	0.0043	0.0043	0.0037	0.0037	0.0030	0.0045	0.0050	0.0048	0.0044	0.0051	0.0050	0.0054
**5**	12	0.0041	0.0038	0.0047	0.0039	0.0037	0.0041	0.0040	0.0044	0.0044	0.0049	0.0048	0.0044	0.0050	0.0050	0.0053
**5**	13	0.0042	0.0037	0.0033	0.0047	0.0047	0.0043	0.0043	0.0043	0.0042	0.0048	0.0047	0.0043	0.0050	0.0048	0.0051
**5**	14	0.0042	0.0040	0.0045	0.0046	0.0045	0.0040	0.0041	0.0042	0.0043	0.0044	0.0047	0.0043	0.0049	0.0048	0.0049
**5**	15	0.0047	0.0045	0.0042	0.0043	0.0041	0.0040	0.0038	0.0043	0.0043	0.0046	0.0044	0.0041	0.0050	0.0049	0.0052
**6**	1	0.0040	0.0035	0.0046	0.0045	0.0045	0.0044	0.0044	0.0046	0.0048	0.0043	0.0044	0.0043	0.0046	0.0046	0.0053
**6**	2	0.0047	0.0046	0.0040	0.0041	0.0041	0.0036	0.0036	0.0043	0.0051	0.0051	0.0043	0.0044	0.0047	0.0047	0.0052
**6**	3	0.0054	0.0052	0.0047	0.0044	0.0044	0.0048	0.0048	0.0042	0.0047	0.0043	0.0039	0.0039	0.0036	0.0038	0.0046
**6**	4	0.0039	0.0036	0.0044	0.0049	0.0049	0.0048	0.0048	0.0042	0.0044	0.0043	0.0045	0.0042	0.0045	0.0045	0.0046
**6**	5	0.0052	0.0048	0.0046	0.0045	0.0045	0.0040	0.0040	0.0041	0.0041	0.0044	0.0044	0.0039	0.0045	0.0044	0.0053
**6**	7	0.0046	0.0044	0.0038	0.0048	0.0048	0.0047	0.0047	0.0044	0.0044	0.0035	0.0042	0.0041	0.0046	0.0046	0.0051
**6**	8	0.0049	0.0040	0.0041	0.0045	0.0045	0.0039	0.0039	0.0050	0.0040	0.0045	0.0046	0.0046	0.0046	0.0045	0.0054
**6**	9	0.0045	0.0043	0.0037	0.0048	0.0048	0.0046	0.0046	0.0043	0.0045	0.0041	0.0042	0.0041	0.0045	0.0045	0.0051
**6**	10	0.0041	0.0047	0.0045	0.0048	0.0047	0.0045	0.0047	0.0050	0.0047	0.0026	0.0042	0.0036	0.0047	0.0047	0.0053
**6**	11	0.0044	0.0042	0.0049	0.0043	0.0043	0.0039	0.0039	0.0033	0.0049	0.0047	0.0047	0.0046	0.0046	0.0046	0.0053
**6**	12	0.0039	0.0036	0.0048	0.0039	0.0038	0.0044	0.0043	0.0048	0.0049	0.0046	0.0048	0.0046	0.0045	0.0046	0.0051

**6**	13	0.0040	0.0035	0.0034	0.0048	0.0048	0.0047	0.0047	0.0047	0.0046	0.0045	0.0046	0.0045	0.0045	0.0044	0.0049
**6**	14	0.0040	0.0038	0.0047	0.0047	0.0046	0.0043	0.0044	0.0046	0.0048	0.0041	0.0046	0.0045	0.0044	0.0044	0.0048
**6**	15	0.0045	0.0043	0.0044	0.0044	0.0042	0.0043	0.0041	0.0047	0.0047	0.0043	0.0044	0.0043	0.0045	0.0045	0.0051
**7**	1	0.0037	0.0033	0.0037	0.0048	0.0048	0.0048	0.0048	0.0044	0.0046	0.0037	0.0043	0.0043	0.0051	0.0051	0.0053
**7**	2	0.0043	0.0044	0.0032	0.0044	0.0044	0.0040	0.0040	0.0041	0.0049	0.0044	0.0043	0.0044	0.0053	0.0053	0.0052
**7**	3	0.0050	0.0050	0.0037	0.0047	0.0047	0.0052	0.0052	0.0040	0.0045	0.0037	0.0038	0.0040	0.0041	0.0042	0.0046
**7**	4	0.0036	0.0035	0.0035	0.0052	0.0052	0.0052	0.0052	0.0040	0.0043	0.0037	0.0045	0.0043	0.0050	0.0050	0.0046
**7**	5	0.0048	0.0046	0.0037	0.0048	0.0048	0.0044	0.0044	0.0040	0.0040	0.0038	0.0043	0.0040	0.0051	0.0050	0.0053
**7**	6	0.0046	0.0044	0.0038	0.0048	0.0048	0.0047	0.0047	0.0044	0.0044	0.0035	0.0042	0.0041	0.0046	0.0046	0.0051
**7**	8	0.0045	0.0038	0.0033	0.0047	0.0047	0.0043	0.0043	0.0048	0.0038	0.0038	0.0045	0.0046	0.0051	0.0050	0.0053
**7**	9	0.0041	0.0041	0.0030	0.0051	0.0051	0.0051	0.0051	0.0041	0.0043	0.0035	0.0041	0.0041	0.0051	0.0051	0.0051
**7**	10	0.0038	0.0045	0.0036	0.0051	0.0050	0.0050	0.0051	0.0048	0.0045	0.0023	0.0041	0.0036	0.0053	0.0053	0.0053
**7**	11	0.0040	0.0040	0.0039	0.0046	0.0046	0.0043	0.0043	0.0031	0.0048	0.0040	0.0047	0.0047	0.0052	0.0052	0.0053
**7**	12	0.0036	0.0035	0.0039	0.0042	0.0040	0.0048	0.0047	0.0046	0.0047	0.0039	0.0047	0.0047	0.0050	0.0051	0.0051
**7**	13	0.0037	0.0034	0.0027	0.0051	0.0051	0.0051	0.0051	0.0045	0.0044	0.0039	0.0045	0.0046	0.0050	0.0049	0.0049
**7**	14	0.0037	0.0037	0.0038	0.0050	0.0049	0.0047	0.0048	0.0044	0.0046	0.0035	0.0046	0.0046	0.0050	0.0049	0.0048
**7**	15	0.0041	0.0041	0.0035	0.0047	0.0044	0.0047	0.0044	0.0046	0.0046	0.0037	0.0043	0.0043	0.0051	0.0051	0.0051
**8**	1	0.0040	0.0030	0.0040	0.0044	0.0044	0.0040	0.0040	0.0051	0.0042	0.0047	0.0047	0.0048	0.0051	0.0050	0.0055
**8**	2	0.0046	0.0041	0.0035	0.0041	0.0041	0.0033	0.0033	0.0048	0.0045	0.0056	0.0046	0.0049	0.0053	0.0052	0.0054
**8**	3	0.0054	0.0046	0.0040	0.0044	0.0044	0.0043	0.0043	0.0046	0.0041	0.0047	0.0041	0.0044	0.0040	0.0041	0.0048
**8**	4	0.0039	0.0032	0.0038	0.0048	0.0048	0.0043	0.0043	0.0046	0.0039	0.0047	0.0048	0.0047	0.0050	0.0049	0.0048
**8**	5	0.0051	0.0042	0.0040	0.0044	0.0044	0.0036	0.0036	0.0046	0.0036	0.0048	0.0047	0.0044	0.0051	0.0049	0.0055
**8**	6	0.0049	0.0040	0.0041	0.0045	0.0045	0.0039	0.0039	0.0050	0.0040	0.0045	0.0046	0.0046	0.0046	0.0045	0.0054
**8**	7	0.0045	0.0038	0.0033	0.0047	0.0047	0.0043	0.0043	0.0048	0.0038	0.0038	0.0045	0.0046	0.0051	0.0050	0.0053
**8**	9	0.0045	0.0038	0.0032	0.0047	0.0047	0.0042	0.0042	0.0048	0.0040	0.0045	0.0045	0.0046	0.0051	0.0049	0.0053
**8**	10	0.0041	0.0041	0.0039	0.0047	0.0046	0.0041	0.0042	0.0055	0.0041	0.0029	0.0044	0.0040	0.0053	0.0052	0.0055
**8**	11	0.0043	0.0037	0.0042	0.0042	0.0042	0.0036	0.0036	0.0036	0.0044	0.0051	0.0050	0.0052	0.0052	0.0050	0.0055
**8**	12	0.0039	0.0032	0.0042	0.0038	0.0037	0.0040	0.0039	0.0053	0.0043	0.0050	0.0051	0.0052	0.0050	0.0050	0.0053
**8**	13	0.0040	0.0031	0.0029	0.0047	0.0047	0.0042	0.0042	0.0052	0.0041	0.0050	0.0049	0.0051	0.0050	0.0048	0.0051
**8**	14	0.0039	0.0034	0.0040	0.0046	0.0045	0.0039	0.0040	0.0051	0.0042	0.0045	0.0049	0.0051	0.0050	0.0048	0.0050

**8**	15	0.0044	0.0038	0.0038	0.0043	0.0041	0.0039	0.0037	0.0053	0.0042	0.0047	0.0047	0.0048	0.0050	0.0049	0.0053
**9**	1	0.0036	0.0033	0.0036	0.0047	0.0047	0.0047	0.0047	0.0044	0.0048	0.0043	0.0043	0.0043	0.0050	0.0050	0.0052
**9**	2	0.0042	0.0044	0.0032	0.0043	0.0043	0.0039	0.0039	0.0041	0.0050	0.0051	0.0042	0.0044	0.0052	0.0052	0.0051
**9**	3	0.0049	0.0049	0.0037	0.0047	0.0047	0.0052	0.0052	0.0039	0.0047	0.0043	0.0038	0.0039	0.0040	0.0042	0.0045
**9**	4	0.0036	0.0034	0.0035	0.0052	0.0052	0.0052	0.0052	0.0040	0.0044	0.0043	0.0044	0.0042	0.0050	0.0050	0.0045
**9**	5	0.0047	0.0045	0.0036	0.0047	0.0047	0.0043	0.0043	0.0039	0.0041	0.0044	0.0043	0.0039	0.0050	0.0049	0.0052
**9**	6	0.0045	0.0043	0.0037	0.0048	0.0048	0.0046	0.0046	0.0043	0.0045	0.0041	0.0042	0.0041	0.0045	0.0045	0.0051
**9**	7	0.0041	0.0041	0.0030	0.0051	0.0051	0.0051	0.0051	0.0041	0.0043	0.0035	0.0041	0.0041	0.0051	0.0051	0.0051
**9**	8	0.0045	0.0038	0.0032	0.0047	0.0047	0.0042	0.0042	0.0048	0.0040	0.0045	0.0045	0.0046	0.0051	0.0049	0.0053
**9**	10	0.0038	0.0044	0.0036	0.0050	0.0049	0.0049	0.0050	0.0047	0.0046	0.0026	0.0041	0.0036	0.0052	0.0052	0.0052
**9**	11	0.0040	0.0040	0.0038	0.0045	0.0045	0.0042	0.0042	0.0031	0.0049	0.0047	0.0046	0.0046	0.0051	0.0051	0.0052
**9**	12	0.0036	0.0034	0.0038	0.0041	0.0040	0.0048	0.0046	0.0046	0.0049	0.0046	0.0046	0.0046	0.0050	0.0051	0.0051
**9**	13	0.0036	0.0033	0.0027	0.0050	0.0050	0.0050	0.0050	0.0045	0.0046	0.0045	0.0045	0.0045	0.0049	0.0048	0.0048
**9**	14	0.0036	0.0036	0.0037	0.0049	0.0048	0.0046	0.0047	0.0044	0.0047	0.0041	0.0045	0.0045	0.0049	0.0048	0.0047
**9**	15	0.0041	0.0041	0.0035	0.0046	0.0044	0.0046	0.0044	0.0045	0.0047	0.0044	0.0043	0.0043	0.0050	0.0050	0.0050
**10**	1	0.0033	0.0036	0.0044	0.0047	0.0046	0.0046	0.0047	0.0051	0.0049	0.0028	0.0043	0.0038	0.0053	0.0053	0.0055
**10**	2	0.0039	0.0047	0.0038	0.0043	0.0042	0.0038	0.0039	0.0047	0.0052	0.0033	0.0042	0.0038	0.0055	0.0055	0.0053
**10**	3	0.0045	0.0053	0.0045	0.0047	0.0046	0.0051	0.0052	0.0046	0.0048	0.0028	0.0038	0.0035	0.0042	0.0044	0.0047
**10**	4	0.0033	0.0037	0.0042	0.0052	0.0050	0.0050	0.0052	0.0046	0.0046	0.0028	0.0044	0.0037	0.0052	0.0052	0.0047
**10**	5	0.0043	0.0049	0.0044	0.0047	0.0046	0.0042	0.0043	0.0045	0.0042	0.0028	0.0042	0.0034	0.0053	0.0052	0.0055
**10**	6	0.0041	0.0047	0.0045	0.0048	0.0047	0.0045	0.0047	0.0050	0.0047	0.0026	0.0042	0.0036	0.0047	0.0047	0.0053
**10**	7	0.0038	0.0045	0.0036	0.0051	0.0050	0.0050	0.0051	0.0048	0.0045	0.0023	0.0041	0.0036	0.0053	0.0053	0.0053
**10**	8	0.0041	0.0041	0.0039	0.0047	0.0046	0.0041	0.0042	0.0055	0.0041	0.0029	0.0044	0.0040	0.0053	0.0052	0.0055
**10**	9	0.0038	0.0044	0.0036	0.0050	0.0049	0.0049	0.0050	0.0047	0.0046	0.0026	0.0041	0.0036	0.0052	0.0052	0.0052
**10**	11	0.0037	0.0043	0.0046	0.0045	0.0044	0.0041	0.0042	0.0036	0.0051	0.0030	0.0046	0.0041	0.0053	0.0053	0.0054
**10**	12	0.0033	0.0037	0.0046	0.0041	0.0039	0.0047	0.0047	0.0053	0.0050	0.0029	0.0046	0.0041	0.0052	0.0053	0.0053
**10**	13	0.0034	0.0036	0.0032	0.0050	0.0049	0.0049	0.0050	0.0052	0.0047	0.0029	0.0044	0.0040	0.0052	0.0051	0.0051
**10**	14	0.0033	0.0039	0.0045	0.0049	0.0047	0.0045	0.0047	0.0050	0.0049	0.0026	0.0045	0.0040	0.0051	0.0050	0.0049
**10**	15	0.0038	0.0044	0.0042	0.0046	0.0043	0.0045	0.0044	0.0052	0.0049	0.0028	0.0042	0.0037	0.0052	0.0052	0.0052
**11**	1	0.0035	0.0032	0.0047	0.0043	0.0043	0.0040	0.0040	0.0033	0.0052	0.0049	0.0048	0.0048	0.0051	0.0051	0.0054

**11**	2	0.0041	0.0042	0.0041	0.0039	0.0039	0.0033	0.0033	0.0031	0.0055	0.0059	0.0048	0.0049	0.0053	0.0053	0.0053
**11**	3	0.0048	0.0048	0.0048	0.0042	0.0042	0.0044	0.0044	0.0030	0.0051	0.0050	0.0043	0.0045	0.0041	0.0043	0.0047
**11**	4	0.0035	0.0033	0.0045	0.0047	0.0047	0.0044	0.0044	0.0030	0.0048	0.0050	0.0050	0.0047	0.0051	0.0051	0.0047
**11**	5	0.0046	0.0044	0.0047	0.0043	0.0043	0.0037	0.0037	0.0030	0.0045	0.0050	0.0048	0.0044	0.0051	0.0050	0.0054
**11**	6	0.0044	0.0042	0.0049	0.0043	0.0043	0.0039	0.0039	0.0033	0.0049	0.0047	0.0047	0.0046	0.0046	0.0046	0.0053
**11**	7	0.0040	0.0040	0.0039	0.0046	0.0046	0.0043	0.0043	0.0031	0.0048	0.0040	0.0047	0.0047	0.0052	0.0052	0.0053
**11**	8	0.0043	0.0037	0.0042	0.0042	0.0042	0.0036	0.0036	0.0036	0.0044	0.0051	0.0050	0.0052	0.0052	0.0050	0.0055
**11**	9	0.0040	0.0040	0.0038	0.0045	0.0045	0.0042	0.0042	0.0031	0.0049	0.0047	0.0046	0.0046	0.0051	0.0051	0.0052
**11**	10	0.0037	0.0043	0.0046	0.0045	0.0044	0.0041	0.0042	0.0036	0.0051	0.0030	0.0046	0.0041	0.0053	0.0053	0.0054
**11**	12	0.0035	0.0033	0.0050	0.0037	0.0036	0.0040	0.0039	0.0035	0.0053	0.0052	0.0052	0.0052	0.0051	0.0052	0.0052
**11**	13	0.0035	0.0032	0.0035	0.0045	0.0045	0.0043	0.0043	0.0034	0.0050	0.0052	0.0050	0.0051	0.0050	0.0049	0.0050
**11**	14	0.0035	0.0035	0.0048	0.0044	0.0044	0.0039	0.0040	0.0033	0.0052	0.0047	0.0051	0.0051	0.0050	0.0049	0.0049
**11**	15	0.0040	0.0040	0.0045	0.0042	0.0040	0.0039	0.0037	0.0034	0.0052	0.0050	0.0048	0.0048	0.0051	0.0051	0.0052
**12**	1	0.0032	0.0027	0.0047	0.0039	0.0037	0.0045	0.0044	0.0049	0.0052	0.0048	0.0049	0.0049	0.0050	0.0051	0.0053
**12**	2	0.0037	0.0036	0.0041	0.0036	0.0034	0.0037	0.0036	0.0046	0.0055	0.0057	0.0048	0.0050	0.0052	0.0053	0.0052
**12**	3	0.0043	0.0041	0.0048	0.0038	0.0037	0.0049	0.0048	0.0044	0.0051	0.0049	0.0043	0.0045	0.0040	0.0042	0.0046
**12**	4	0.0031	0.0028	0.0045	0.0042	0.0041	0.0049	0.0048	0.0045	0.0048	0.0048	0.0050	0.0048	0.0049	0.0050	0.0046
**12**	5	0.0041	0.0038	0.0047	0.0039	0.0037	0.0041	0.0040	0.0044	0.0044	0.0049	0.0048	0.0044	0.0050	0.0050	0.0053
**12**	6	0.0039	0.0036	0.0048	0.0039	0.0038	0.0044	0.0043	0.0048	0.0049	0.0046	0.0048	0.0046	0.0045	0.0046	0.0051
**12**	7	0.0036	0.0035	0.0039	0.0042	0.0040	0.0048	0.0047	0.0046	0.0047	0.0039	0.0047	0.0047	0.0050	0.0051	0.0051
**12**	8	0.0039	0.0032	0.0042	0.0038	0.0037	0.0040	0.0039	0.0053	0.0043	0.0050	0.0051	0.0052	0.0050	0.0050	0.0053
**12**	9	0.0036	0.0034	0.0038	0.0041	0.0040	0.0048	0.0046	0.0046	0.0049	0.0046	0.0046	0.0046	0.0050	0.0051	0.0051
**12**	10	0.0033	0.0037	0.0046	0.0041	0.0039	0.0047	0.0047	0.0053	0.0050	0.0029	0.0046	0.0041	0.0052	0.0053	0.0053
**12**	11	0.0035	0.0033	0.0050	0.0037	0.0036	0.0040	0.0039	0.0035	0.0053	0.0052	0.0052	0.0052	0.0051	0.0052	0.0052
**12**	13	0.0032	0.0028	0.0035	0.0041	0.0040	0.0048	0.0047	0.0050	0.0050	0.0051	0.0051	0.0052	0.0049	0.0049	0.0049
**12**	14	0.0032	0.0030	0.0048	0.0040	0.0038	0.0044	0.0044	0.0049	0.0051	0.0046	0.0051	0.0051	0.0049	0.0049	0.0048
**12**	15	0.0036	0.0034	0.0045	0.0038	0.0035	0.0044	0.0041	0.0050	0.0051	0.0049	0.0048	0.0048	0.0049	0.0050	0.0050
**13**	1	0.0032	0.0027	0.0033	0.0048	0.0048	0.0048	0.0048	0.0048	0.0049	0.0048	0.0047	0.0048	0.0050	0.0049	0.0051
**13**	2	0.0038	0.0036	0.0029	0.0044	0.0044	0.0039	0.0039	0.0045	0.0052	0.0057	0.0046	0.0049	0.0052	0.0051	0.0049
**13**	3	0.0044	0.0040	0.0034	0.0047	0.0047	0.0052	0.0052	0.0043	0.0048	0.0048	0.0041	0.0044	0.0039	0.0041	0.0044

**13**	4	0.0032	0.0028	0.0032	0.0052	0.0052	0.0052	0.0052	0.0044	0.0045	0.0048	0.0048	0.0047	0.0049	0.0048	0.0044
**13**	5	0.0042	0.0037	0.0033	0.0047	0.0047	0.0043	0.0043	0.0043	0.0042	0.0048	0.0047	0.0043	0.0050	0.0048	0.0051
**13**	6	0.0040	0.0035	0.0034	0.0048	0.0048	0.0047	0.0047	0.0047	0.0046	0.0045	0.0046	0.0045	0.0045	0.0044	0.0049
**13**	7	0.0037	0.0034	0.0027	0.0051	0.0051	0.0051	0.0051	0.0045	0.0044	0.0039	0.0045	0.0046	0.0050	0.0049	0.0049
**13**	8	0.0040	0.0031	0.0029	0.0047	0.0047	0.0042	0.0042	0.0052	0.0041	0.0050	0.0049	0.0051	0.0050	0.0048	0.0051
**13**	9	0.0036	0.0033	0.0027	0.0050	0.0050	0.0050	0.0050	0.0045	0.0046	0.0045	0.0045	0.0045	0.0049	0.0048	0.0048
**13**	10	0.0034	0.0036	0.0032	0.0050	0.0049	0.0049	0.0050	0.0052	0.0047	0.0029	0.0044	0.0040	0.0052	0.0051	0.0051
**13**	11	0.0035	0.0032	0.0035	0.0045	0.0045	0.0043	0.0043	0.0034	0.0050	0.0052	0.0050	0.0051	0.0050	0.0049	0.0050
**13**	12	0.0032	0.0028	0.0035	0.0041	0.0040	0.0048	0.0047	0.0050	0.0050	0.0051	0.0051	0.0052	0.0049	0.0049	0.0049
**13**	14	0.0032	0.0029	0.0034	0.0049	0.0048	0.0046	0.0048	0.0048	0.0048	0.0045	0.0049	0.0050	0.0049	0.0047	0.0046
**13**	15	0.0036	0.0033	0.0031	0.0046	0.0044	0.0046	0.0044	0.0049	0.0048	0.0048	0.0047	0.0047	0.0049	0.0048	0.0048
**14**	1	0.0032	0.0029	0.0046	0.0046	0.0046	0.0044	0.0045	0.0047	0.0050	0.0043	0.0047	0.0047	0.0050	0.0049	0.0049
**14**	2	0.0037	0.0039	0.0040	0.0043	0.0042	0.0036	0.0037	0.0044	0.0053	0.0051	0.0047	0.0048	0.0051	0.0050	0.0048
**14**	3	0.0044	0.0044	0.0046	0.0046	0.0045	0.0048	0.0049	0.0042	0.0049	0.0043	0.0042	0.0044	0.0039	0.0040	0.0043
**14**	4	0.0032	0.0030	0.0044	0.0051	0.0050	0.0048	0.0049	0.0043	0.0047	0.0043	0.0049	0.0047	0.0049	0.0048	0.0043
**14**	5	0.0042	0.0040	0.0045	0.0046	0.0045	0.0040	0.0041	0.0042	0.0043	0.0044	0.0047	0.0043	0.0049	0.0048	0.0049
**14**	6	0.0040	0.0038	0.0047	0.0047	0.0046	0.0043	0.0044	0.0046	0.0048	0.0041	0.0046	0.0045	0.0044	0.0044	0.0048
**14**	7	0.0037	0.0037	0.0038	0.0050	0.0049	0.0047	0.0048	0.0044	0.0046	0.0035	0.0046	0.0046	0.0050	0.0049	0.0048
**14**	8	0.0039	0.0034	0.0040	0.0046	0.0045	0.0039	0.0040	0.0051	0.0042	0.0045	0.0049	0.0051	0.0050	0.0048	0.0050
**14**	9	0.0036	0.0036	0.0037	0.0049	0.0048	0.0046	0.0047	0.0044	0.0047	0.0041	0.0045	0.0045	0.0049	0.0048	0.0047
**14**	10	0.0033	0.0039	0.0045	0.0049	0.0047	0.0045	0.0047	0.0050	0.0049	0.0026	0.0045	0.0040	0.0051	0.0050	0.0049
**14**	11	0.0035	0.0035	0.0048	0.0044	0.0044	0.0039	0.0040	0.0033	0.0052	0.0047	0.0051	0.0051	0.0050	0.0049	0.0049
**14**	12	0.0032	0.0030	0.0048	0.0040	0.0038	0.0044	0.0044	0.0049	0.0051	0.0046	0.0051	0.0051	0.0049	0.0049	0.0048
**14**	13	0.0032	0.0029	0.0034	0.0049	0.0048	0.0046	0.0048	0.0048	0.0048	0.0045	0.0049	0.0050	0.0049	0.0047	0.0046
**14**	15	0.0036	0.0036	0.0043	0.0045	0.0042	0.0043	0.0042	0.0048	0.0050	0.0043	0.0047	0.0047	0.0049	0.0048	0.0047
**15**	1	0.0036	0.0033	0.0043	0.0044	0.0042	0.0044	0.0042	0.0048	0.0050	0.0046	0.0045	0.0045	0.0050	0.0050	0.0052
**15**	2	0.0042	0.0044	0.0037	0.0040	0.0038	0.0036	0.0034	0.0045	0.0053	0.0054	0.0044	0.0046	0.0052	0.0052	0.0051
**15**	3	0.0049	0.0049	0.0043	0.0043	0.0041	0.0048	0.0045	0.0044	0.0049	0.0046	0.0040	0.0041	0.0040	0.0042	0.0045
**15**	4	0.0036	0.0034	0.0041	0.0048	0.0045	0.0048	0.0045	0.0044	0.0046	0.0046	0.0046	0.0044	0.0050	0.0050	0.0045
**15**	5	0.0047	0.0045	0.0042	0.0043	0.0041	0.0040	0.0038	0.0043	0.0043	0.0046	0.0044	0.0041	0.0050	0.0049	0.0052

**15**	6	0.0045	0.0043	0.0044	0.0044	0.0042	0.0043	0.0041	0.0047	0.0047	0.0043	0.0044	0.0043	0.0045	0.0045	0.0051
**15**	7	0.0041	0.0041	0.0035	0.0047	0.0044	0.0047	0.0044	0.0046	0.0046	0.0037	0.0043	0.0043	0.0051	0.0051	0.0051
**15**	8	0.0044	0.0038	0.0038	0.0043	0.0041	0.0039	0.0037	0.0053	0.0042	0.0047	0.0047	0.0048	0.0050	0.0049	0.0053
**15**	9	0.0041	0.0041	0.0035	0.0046	0.0044	0.0046	0.0044	0.0045	0.0047	0.0044	0.0043	0.0043	0.0050	0.0050	0.0050
**15**	10	0.0038	0.0044	0.0042	0.0046	0.0043	0.0045	0.0044	0.0052	0.0049	0.0028	0.0042	0.0037	0.0052	0.0052	0.0052
**15**	11	0.0040	0.0040	0.0045	0.0042	0.0040	0.0039	0.0037	0.0034	0.0052	0.0050	0.0048	0.0048	0.0051	0.0051	0.0052
**15**	12	0.0036	0.0034	0.0045	0.0038	0.0035	0.0044	0.0041	0.0050	0.0051	0.0049	0.0048	0.0048	0.0049	0.0050	0.0050
**15**	13	0.0036	0.0033	0.0031	0.0046	0.0044	0.0046	0.0044	0.0049	0.0048	0.0048	0.0047	0.0047	0.0049	0.0048	0.0048
**15**	14	0.0036	0.0036	0.0043	0.0045	0.0042	0.0043	0.0042	0.0048	0.0050	0.0043	0.0047	0.0047	0.0049	0.0048	0.0047

Table S4 Initial matrix obtained expert scores for denim pant

	**C1**	**C2**	**C3**	**C4**	**C5**	**C6**	**C7**	**C8**	**C9**	**C10**	**C11**	**C12**	**C13**	**C14**	**C15**
**E1**	10	8	20	15	15	15	15	16	18	15	20	20	18	18	20
**E2**	10	10	9	14	13	10	10	12	20	15	14	16	16	16	17
**E3**	19	18	14	15	15	18	18	13	16	12	13	14	9	10	12
**E4**	10	9	16	20	20	20	20	13	19	16	17	15	18	18	14
**E5**	20	18	18	18	18	16	16	12	16	12	15	12	18	20	20
**E6**	15	15	15	15	15	14	14	13	15	17	15	15	13	13	18
**E7**	15	15	10	20	20	20	20	15	15	10	15	15	20	20	20
**E8**	15	18	17	19	19	19	19	18	18	10	19	20	16	15	18
**E9**	15	15	15	20	20	20	20	15	20	15	15	15	20	20	20
**E10**	15	18	10	16	15	17	17	10	18	5	14	12	16	16	18
**E11**	9	10	15	13	13	11	11	7	19	17	18	18	18	18	19
**E12**	11	10	19	10	11	15	16	18	20	18	20	20	19	18	19
**E13**	10	10	8	20	20	20	20	19	20	16	18	19	16	18	18
**E14**	10	11	18	20	18	17	16	18	20	15	20	20	19	18	16
**E15**	15	15	15	16	14	16	14	20	20	18	17	17	20	20	20

Table S5 Weights for each expert for denim pant using Equation 1

	**C1**	**C2**	**C3**	**C4**	**C5**	**C6**	**C7**	**C8**	**C9**	**C10**	**C11**	**C12**	**C13**	**C14**	**C15**

**1**	0.0557	0.0503	0.0725	0.0655	0.0655	0.0655	0.0655	0.0671	0.0699	0.0655	0.0725	0.0725	0.0699	0.0699	0.0725
**2**	0.0597	0.0597	0.0569	0.0684	0.0665	0.0597	0.0597	0.0644	0.0776	0.0702	0.0684	0.0718	0.0718	0.0718	0.0734
**3**	0.0742	0.0728	0.0665	0.0682	0.0682	0.0728	0.0728	0.0646	0.0699	0.0626	0.0646	0.0665	0.0554	0.0580	0.0626
**4**	0.0555	0.0529	0.0668	0.0722	0.0722	0.0722	0.0722	0.0618	0.0710	0.0668	0.0683	0.0653	0.0697	0.0697	0.0636
**5**	0.0715	0.0690	0.0690	0.0690	0.0690	0.0661	0.0661	0.0593	0.0661	0.0593	0.0646	0.0593	0.0690	0.0715	0.0715
**6**	0.0671	0.0671	0.0671	0.0671	0.0671	0.0654	0.0654	0.0636	0.0671	0.0702	0.0671	0.0671	0.0636	0.0636	0.0716
**7**	0.0647	0.0647	0.0551	0.0716	0.0716	0.0716	0.0716	0.0647	0.0647	0.0551	0.0647	0.0647	0.0716	0.0716	0.0716
**8**	0.0636	0.0678	0.0665	0.0691	0.0691	0.0691	0.0691	0.0678	0.0678	0.0540	0.0691	0.0703	0.0651	0.0636	0.0678
**9**	0.0631	0.0631	0.0631	0.0698	0.0698	0.0698	0.0698	0.0631	0.0698	0.0631	0.0631	0.0631	0.0698	0.0698	0.0698
**10**	0.0687	0.0733	0.0584	0.0704	0.0687	0.0719	0.0719	0.0584	0.0733	0.0408	0.0670	0.0631	0.0704	0.0704	0.0733
**11**	0.0558	0.0585	0.0688	0.0652	0.0652	0.0609	0.0609	0.0494	0.0748	0.0720	0.0734	0.0734	0.0734	0.0734	0.0748
**12**	0.0580	0.0557	0.0712	0.0557	0.0580	0.0655	0.0670	0.0699	0.0724	0.0699	0.0724	0.0724	0.0712	0.0699	0.0712
**13**	0.0551	0.0551	0.0498	0.0717	0.0717	0.0717	0.0717	0.0705	0.0717	0.0664	0.0692	0.0705	0.0664	0.0692	0.0692
**14**	0.0545	0.0567	0.0684	0.0709	0.0684	0.0670	0.0656	0.0684	0.0709	0.0641	0.0709	0.0709	0.0696	0.0684	0.0656
**15**	0.0637	0.0637	0.0637	0.0653	0.0621	0.0653	0.0621	0.0705	0.0705	0.0680	0.0667	0.0667	0.0705	0.0705	0.0705

Table S6 Aggregated weights for denim pant using Equation 2

**E**	**E**	**C1**	**C2**	**C3**	**C4**	**C5**	**C6**	**C7**	**C8**	**C9**	**C10**	**C11**	**C12**	**C13**	**C14**	**C15**
**1**	2	0.0033	0.0030	0.0041	0.0045	0.0044	0.0039	0.0039	0.0043	0.0054	0.0046	0.0050	0.0052	0.0050	0.0050	0.0053
**1**	3	0.0041	0.0037	0.0048	0.0045	0.0045	0.0048	0.0048	0.0043	0.0049	0.0041	0.0047	0.0048	0.0039	0.0041	0.0045
**1**	4	0.0031	0.0027	0.0048	0.0047	0.0047	0.0047	0.0047	0.0041	0.0050	0.0044	0.0049	0.0047	0.0049	0.0049	0.0046
**1**	5	0.0040	0.0035	0.0050	0.0045	0.0045	0.0043	0.0043	0.0040	0.0046	0.0039	0.0047	0.0043	0.0048	0.0050	0.0052
**1**	6	0.0037	0.0034	0.0049	0.0044	0.0044	0.0043	0.0043	0.0043	0.0047	0.0046	0.0049	0.0049	0.0044	0.0044	0.0052
**1**	7	0.0036	0.0033	0.0040	0.0047	0.0047	0.0047	0.0047	0.0043	0.0045	0.0036	0.0047	0.0047	0.0050	0.0050	0.0052
**1**	8	0.0035	0.0034	0.0048	0.0045	0.0045	0.0045	0.0045	0.0045	0.0047	0.0035	0.0050	0.0051	0.0045	0.0044	0.0049
**1**	9	0.0035	0.0032	0.0046	0.0046	0.0046	0.0046	0.0046	0.0042	0.0049	0.0041	0.0046	0.0046	0.0049	0.0049	0.0051
**1**	10	0.0038	0.0037	0.0042	0.0046	0.0045	0.0047	0.0047	0.0039	0.0051	0.0027	0.0049	0.0046	0.0049	0.0049	0.0053
**1**	11	0.0031	0.0029	0.0050	0.0043	0.0043	0.0040	0.0040	0.0033	0.0052	0.0047	0.0053	0.0053	0.0051	0.0051	0.0054
**1**	12	0.0032	0.0028	0.0052	0.0036	0.0038	0.0043	0.0044	0.0047	0.0051	0.0046	0.0052	0.0052	0.0050	0.0049	0.0052
**1**	13	0.0031	0.0028	0.0036	0.0047	0.0047	0.0047	0.0047	0.0047	0.0050	0.0043	0.0050	0.0051	0.0046	0.0048	0.0050

**1**	14	0.0030	0.0029	0.0050	0.0046	0.0045	0.0044	0.0043	0.0046	0.0050	0.0042	0.0051	0.0051	0.0049	0.0048	0.0048
**1**	15	0.0035	0.0032	0.0046	0.0043	0.0041	0.0043	0.0041	0.0047	0.0049	0.0045	0.0048	0.0048	0.0049	0.0049	0.0051
**2**	1	0.0033	0.0030	0.0041	0.0045	0.0044	0.0039	0.0039	0.0043	0.0054	0.0046	0.0050	0.0052	0.0050	0.0050	0.0053
**2**	3	0.0044	0.0043	0.0038	0.0047	0.0045	0.0043	0.0043	0.0042	0.0054	0.0044	0.0044	0.0048	0.0040	0.0042	0.0046
**2**	4	0.0033	0.0032	0.0038	0.0049	0.0048	0.0043	0.0043	0.0040	0.0055	0.0047	0.0047	0.0047	0.0050	0.0050	0.0047
**2**	5	0.0043	0.0041	0.0039	0.0047	0.0046	0.0039	0.0039	0.0038	0.0051	0.0042	0.0044	0.0043	0.0050	0.0051	0.0052
**2**	6	0.0040	0.0040	0.0038	0.0046	0.0045	0.0039	0.0039	0.0041	0.0052	0.0049	0.0046	0.0048	0.0046	0.0046	0.0053
**2**	7	0.0039	0.0039	0.0031	0.0049	0.0048	0.0043	0.0043	0.0042	0.0050	0.0039	0.0044	0.0047	0.0051	0.0051	0.0053
**2**	8	0.0038	0.0040	0.0038	0.0047	0.0046	0.0041	0.0041	0.0044	0.0053	0.0038	0.0047	0.0051	0.0047	0.0046	0.0050
**2**	9	0.0038	0.0038	0.0036	0.0048	0.0046	0.0042	0.0042	0.0041	0.0054	0.0044	0.0043	0.0045	0.0050	0.0050	0.0051
**2**	10	0.0041	0.0044	0.0033	0.0048	0.0046	0.0043	0.0043	0.0038	0.0057	0.0029	0.0046	0.0045	0.0051	0.0051	0.0054
**2**	11	0.0033	0.0035	0.0039	0.0045	0.0043	0.0036	0.0036	0.0032	0.0058	0.0051	0.0050	0.0053	0.0053	0.0053	0.0055
**2**	12	0.0035	0.0033	0.0041	0.0038	0.0039	0.0039	0.0040	0.0045	0.0056	0.0049	0.0050	0.0052	0.0051	0.0050	0.0052
**2**	13	0.0033	0.0033	0.0028	0.0049	0.0048	0.0043	0.0043	0.0045	0.0056	0.0047	0.0047	0.0051	0.0048	0.0050	0.0051
**2**	14	0.0032	0.0034	0.0039	0.0048	0.0045	0.0040	0.0039	0.0044	0.0055	0.0045	0.0048	0.0051	0.0050	0.0049	0.0048
**2**	15	0.0038	0.0038	0.0036	0.0045	0.0041	0.0039	0.0037	0.0045	0.0055	0.0048	0.0046	0.0048	0.0051	0.0051	0.0052
**3**	1	0.0041	0.0037	0.0048	0.0045	0.0045	0.0048	0.0048	0.0043	0.0049	0.0041	0.0047	0.0048	0.0039	0.0041	0.0045
**3**	2	0.0044	0.0043	0.0038	0.0047	0.0045	0.0043	0.0043	0.0042	0.0054	0.0044	0.0044	0.0048	0.0040	0.0042	0.0046
**3**	4	0.0041	0.0039	0.0044	0.0049	0.0049	0.0053	0.0053	0.0040	0.0050	0.0042	0.0044	0.0043	0.0039	0.0040	0.0040
**3**	5	0.0053	0.0050	0.0046	0.0047	0.0047	0.0048	0.0048	0.0038	0.0046	0.0037	0.0042	0.0039	0.0038	0.0041	0.0045
**3**	6	0.0050	0.0049	0.0045	0.0046	0.0046	0.0048	0.0048	0.0041	0.0047	0.0044	0.0043	0.0045	0.0035	0.0037	0.0045
**3**	7	0.0048	0.0047	0.0037	0.0049	0.0049	0.0052	0.0052	0.0042	0.0045	0.0034	0.0042	0.0043	0.0040	0.0042	0.0045
**3**	8	0.0047	0.0049	0.0044	0.0047	0.0047	0.0050	0.0050	0.0044	0.0047	0.0034	0.0045	0.0047	0.0036	0.0037	0.0042
**3**	9	0.0047	0.0046	0.0042	0.0048	0.0048	0.0051	0.0051	0.0041	0.0049	0.0040	0.0041	0.0042	0.0039	0.0040	0.0044
**3**	10	0.0051	0.0053	0.0039	0.0048	0.0047	0.0052	0.0052	0.0038	0.0051	0.0026	0.0043	0.0042	0.0039	0.0041	0.0046
**3**	11	0.0041	0.0043	0.0046	0.0044	0.0044	0.0044	0.0044	0.0032	0.0052	0.0045	0.0047	0.0049	0.0041	0.0043	0.0047
**3**	12	0.0043	0.0041	0.0047	0.0038	0.0040	0.0048	0.0049	0.0045	0.0051	0.0044	0.0047	0.0048	0.0039	0.0041	0.0045
**3**	13	0.0041	0.0040	0.0033	0.0049	0.0049	0.0052	0.0052	0.0046	0.0050	0.0042	0.0045	0.0047	0.0037	0.0040	0.0043
**3**	14	0.0040	0.0041	0.0045	0.0048	0.0047	0.0049	0.0048	0.0044	0.0050	0.0040	0.0046	0.0047	0.0039	0.0040	0.0041
**3**	15	0.0047	0.0046	0.0042	0.0045	0.0042	0.0048	0.0045	0.0046	0.0049	0.0043	0.0043	0.0044	0.0039	0.0041	0.0044

**4**	1	0.0031	0.0027	0.0048	0.0047	0.0047	0.0047	0.0047	0.0041	0.0050	0.0044	0.0049	0.0047	0.0049	0.0049	0.0046
**4**	2	0.0033	0.0032	0.0038	0.0049	0.0048	0.0043	0.0043	0.0040	0.0055	0.0047	0.0047	0.0047	0.0050	0.0050	0.0047
**4**	3	0.0041	0.0039	0.0044	0.0049	0.0049	0.0053	0.0053	0.0040	0.0050	0.0042	0.0044	0.0043	0.0039	0.0040	0.0040
**4**	5	0.0040	0.0037	0.0046	0.0050	0.0050	0.0048	0.0048	0.0037	0.0047	0.0040	0.0044	0.0039	0.0048	0.0050	0.0045
**4**	6	0.0037	0.0036	0.0045	0.0048	0.0048	0.0047	0.0047	0.0039	0.0048	0.0047	0.0046	0.0044	0.0044	0.0044	0.0046
**4**	7	0.0036	0.0034	0.0037	0.0052	0.0052	0.0052	0.0052	0.0040	0.0046	0.0037	0.0044	0.0042	0.0050	0.0050	0.0046
**4**	8	0.0035	0.0036	0.0044	0.0050	0.0050	0.0050	0.0050	0.0042	0.0048	0.0036	0.0047	0.0046	0.0045	0.0044	0.0043
**4**	9	0.0035	0.0033	0.0042	0.0050	0.0050	0.0050	0.0050	0.0039	0.0050	0.0042	0.0043	0.0041	0.0049	0.0049	0.0044
**4**	10	0.0038	0.0039	0.0039	0.0051	0.0050	0.0052	0.0052	0.0036	0.0052	0.0027	0.0046	0.0041	0.0049	0.0049	0.0047
**4**	11	0.0031	0.0031	0.0046	0.0047	0.0047	0.0044	0.0044	0.0031	0.0053	0.0048	0.0050	0.0048	0.0051	0.0051	0.0048
**4**	12	0.0032	0.0029	0.0048	0.0040	0.0042	0.0047	0.0048	0.0043	0.0051	0.0047	0.0049	0.0047	0.0050	0.0049	0.0045
**4**	13	0.0031	0.0029	0.0033	0.0052	0.0052	0.0052	0.0052	0.0044	0.0051	0.0044	0.0047	0.0046	0.0046	0.0048	0.0044
**4**	14	0.0030	0.0030	0.0046	0.0051	0.0049	0.0048	0.0047	0.0042	0.0050	0.0043	0.0048	0.0046	0.0049	0.0048	0.0042
**4**	15	0.0035	0.0034	0.0043	0.0047	0.0045	0.0047	0.0045	0.0044	0.0050	0.0045	0.0046	0.0044	0.0049	0.0049	0.0045
**5**	1	0.0040	0.0035	0.0050	0.0045	0.0045	0.0043	0.0043	0.0040	0.0046	0.0039	0.0047	0.0043	0.0048	0.0050	0.0052
**5**	2	0.0043	0.0041	0.0039	0.0047	0.0046	0.0039	0.0039	0.0038	0.0051	0.0042	0.0044	0.0043	0.0050	0.0051	0.0052
**5**	3	0.0053	0.0050	0.0046	0.0047	0.0047	0.0048	0.0048	0.0038	0.0046	0.0037	0.0042	0.0039	0.0038	0.0041	0.0045
**5**	4	0.0040	0.0037	0.0046	0.0050	0.0050	0.0048	0.0048	0.0037	0.0047	0.0040	0.0044	0.0039	0.0048	0.0050	0.0045
**5**	6	0.0048	0.0046	0.0046	0.0046	0.0046	0.0043	0.0043	0.0038	0.0044	0.0042	0.0043	0.0040	0.0044	0.0045	0.0051
**5**	7	0.0046	0.0045	0.0038	0.0049	0.0049	0.0047	0.0047	0.0038	0.0043	0.0033	0.0042	0.0038	0.0049	0.0051	0.0051
**5**	8	0.0045	0.0047	0.0046	0.0048	0.0048	0.0046	0.0046	0.0040	0.0045	0.0032	0.0045	0.0042	0.0045	0.0045	0.0048
**5**	9	0.0045	0.0044	0.0044	0.0048	0.0048	0.0046	0.0046	0.0037	0.0046	0.0037	0.0041	0.0037	0.0048	0.0050	0.0050
**5**	10	0.0049	0.0051	0.0040	0.0049	0.0047	0.0048	0.0048	0.0035	0.0049	0.0024	0.0043	0.0037	0.0049	0.0050	0.0052
**5**	11	0.0040	0.0040	0.0047	0.0045	0.0045	0.0040	0.0040	0.0029	0.0049	0.0043	0.0047	0.0044	0.0051	0.0052	0.0053
**5**	12	0.0041	0.0038	0.0049	0.0038	0.0040	0.0043	0.0044	0.0041	0.0048	0.0041	0.0047	0.0043	0.0049	0.0050	0.0051
**5**	13	0.0039	0.0038	0.0034	0.0049	0.0049	0.0047	0.0047	0.0042	0.0047	0.0039	0.0045	0.0042	0.0046	0.0049	0.0049
**5**	14	0.0039	0.0039	0.0047	0.0049	0.0047	0.0044	0.0043	0.0041	0.0047	0.0038	0.0046	0.0042	0.0048	0.0049	0.0047
**5**	15	0.0046	0.0044	0.0044	0.0045	0.0043	0.0043	0.0041	0.0042	0.0047	0.0040	0.0043	0.0040	0.0049	0.0050	0.0050
**6**	1	0.0037	0.0034	0.0049	0.0044	0.0044	0.0043	0.0043	0.0043	0.0047	0.0046	0.0049	0.0049	0.0044	0.0044	0.0052
**6**	2	0.0040	0.0040	0.0038	0.0046	0.0045	0.0039	0.0039	0.0041	0.0052	0.0049	0.0046	0.0048	0.0046	0.0046	0.0053

**6**	3	0.0050	0.0049	0.0045	0.0046	0.0046	0.0048	0.0048	0.0041	0.0047	0.0044	0.0043	0.0045	0.0035	0.0037	0.0045
**6**	4	0.0037	0.0036	0.0045	0.0048	0.0048	0.0047	0.0047	0.0039	0.0048	0.0047	0.0046	0.0044	0.0044	0.0044	0.0046
**6**	5	0.0048	0.0046	0.0046	0.0046	0.0046	0.0043	0.0043	0.0038	0.0044	0.0042	0.0043	0.0040	0.0044	0.0045	0.0051
**6**	7	0.0043	0.0043	0.0037	0.0048	0.0048	0.0047	0.0047	0.0041	0.0043	0.0039	0.0043	0.0043	0.0046	0.0046	0.0051
**6**	8	0.0043	0.0046	0.0045	0.0046	0.0046	0.0045	0.0045	0.0043	0.0046	0.0038	0.0046	0.0047	0.0041	0.0040	0.0049
**6**	9	0.0042	0.0042	0.0042	0.0047	0.0047	0.0046	0.0046	0.0040	0.0047	0.0044	0.0042	0.0042	0.0044	0.0044	0.0050
**6**	10	0.0046	0.0049	0.0039	0.0047	0.0046	0.0047	0.0047	0.0037	0.0049	0.0029	0.0045	0.0042	0.0045	0.0045	0.0053
**6**	11	0.0037	0.0039	0.0046	0.0044	0.0044	0.0040	0.0040	0.0031	0.0050	0.0051	0.0049	0.0049	0.0047	0.0047	0.0054
**6**	12	0.0039	0.0037	0.0048	0.0037	0.0039	0.0043	0.0044	0.0044	0.0049	0.0049	0.0049	0.0049	0.0045	0.0044	0.0051
**6**	13	0.0037	0.0037	0.0033	0.0048	0.0048	0.0047	0.0047	0.0045	0.0048	0.0047	0.0046	0.0047	0.0042	0.0044	0.0050
**6**	14	0.0037	0.0038	0.0046	0.0048	0.0046	0.0044	0.0043	0.0043	0.0048	0.0045	0.0048	0.0048	0.0044	0.0043	0.0047
**6**	15	0.0043	0.0043	0.0043	0.0044	0.0042	0.0043	0.0041	0.0045	0.0047	0.0048	0.0045	0.0045	0.0045	0.0045	0.0050
**7**	1	0.0036	0.0033	0.0040	0.0047	0.0047	0.0047	0.0047	0.0043	0.0045	0.0036	0.0047	0.0047	0.0050	0.0050	0.0052
**7**	2	0.0039	0.0039	0.0031	0.0049	0.0048	0.0043	0.0043	0.0042	0.0050	0.0039	0.0044	0.0047	0.0051	0.0051	0.0053
**7**	3	0.0048	0.0047	0.0037	0.0049	0.0049	0.0052	0.0052	0.0042	0.0045	0.0034	0.0042	0.0043	0.0040	0.0042	0.0045
**7**	4	0.0036	0.0034	0.0037	0.0052	0.0052	0.0052	0.0052	0.0040	0.0046	0.0037	0.0044	0.0042	0.0050	0.0050	0.0046
**7**	5	0.0046	0.0045	0.0038	0.0049	0.0049	0.0047	0.0047	0.0038	0.0043	0.0033	0.0042	0.0038	0.0049	0.0051	0.0051
**7**	6	0.0043	0.0043	0.0037	0.0048	0.0048	0.0047	0.0047	0.0041	0.0043	0.0039	0.0043	0.0043	0.0046	0.0046	0.0051
**7**	8	0.0041	0.0044	0.0037	0.0050	0.0050	0.0050	0.0050	0.0044	0.0044	0.0030	0.0045	0.0046	0.0047	0.0046	0.0049
**7**	9	0.0041	0.0041	0.0035	0.0050	0.0050	0.0050	0.0050	0.0041	0.0045	0.0035	0.0041	0.0041	0.0050	0.0050	0.0050
**7**	10	0.0044	0.0047	0.0032	0.0050	0.0049	0.0051	0.0051	0.0038	0.0047	0.0022	0.0043	0.0041	0.0050	0.0050	0.0053
**7**	11	0.0036	0.0038	0.0038	0.0047	0.0047	0.0044	0.0044	0.0032	0.0048	0.0040	0.0048	0.0048	0.0053	0.0053	0.0054
**7**	12	0.0038	0.0036	0.0039	0.0040	0.0042	0.0047	0.0048	0.0045	0.0047	0.0038	0.0047	0.0047	0.0051	0.0050	0.0051
**7**	13	0.0036	0.0036	0.0027	0.0051	0.0051	0.0051	0.0051	0.0046	0.0046	0.0037	0.0045	0.0046	0.0048	0.0050	0.0050
**7**	14	0.0035	0.0037	0.0038	0.0051	0.0049	0.0048	0.0047	0.0044	0.0046	0.0035	0.0046	0.0046	0.0050	0.0049	0.0047
**7**	15	0.0041	0.0041	0.0035	0.0047	0.0044	0.0047	0.0044	0.0046	0.0046	0.0037	0.0043	0.0043	0.0051	0.0051	0.0051
**8**	1	0.0035	0.0034	0.0048	0.0045	0.0045	0.0045	0.0045	0.0045	0.0047	0.0035	0.0050	0.0051	0.0045	0.0044	0.0049
**8**	2	0.0038	0.0040	0.0038	0.0047	0.0046	0.0041	0.0041	0.0044	0.0053	0.0038	0.0047	0.0051	0.0047	0.0046	0.0050
**8**	3	0.0047	0.0049	0.0044	0.0047	0.0047	0.0050	0.0050	0.0044	0.0047	0.0034	0.0045	0.0047	0.0036	0.0037	0.0042
**8**	4	0.0035	0.0036	0.0044	0.0050	0.0050	0.0050	0.0050	0.0042	0.0048	0.0036	0.0047	0.0046	0.0045	0.0044	0.0043

**8**	5	0.0045	0.0047	0.0046	0.0048	0.0048	0.0046	0.0046	0.0040	0.0045	0.0032	0.0045	0.0042	0.0045	0.0045	0.0048
**8**	6	0.0043	0.0046	0.0045	0.0046	0.0046	0.0045	0.0045	0.0043	0.0046	0.0038	0.0046	0.0047	0.0041	0.0040	0.0049
**8**	7	0.0041	0.0044	0.0037	0.0050	0.0050	0.0050	0.0050	0.0044	0.0044	0.0030	0.0045	0.0046	0.0047	0.0046	0.0049
**8**	9	0.0040	0.0043	0.0042	0.0048	0.0048	0.0048	0.0048	0.0043	0.0047	0.0034	0.0044	0.0044	0.0045	0.0044	0.0047
**8**	10	0.0044	0.0050	0.0039	0.0049	0.0047	0.0050	0.0050	0.0040	0.0050	0.0022	0.0046	0.0044	0.0046	0.0045	0.0050
**8**	11	0.0035	0.0040	0.0046	0.0045	0.0045	0.0042	0.0042	0.0034	0.0051	0.0039	0.0051	0.0052	0.0048	0.0047	0.0051
**8**	12	0.0037	0.0038	0.0047	0.0038	0.0040	0.0045	0.0046	0.0047	0.0049	0.0038	0.0050	0.0051	0.0046	0.0044	0.0048
**8**	13	0.0035	0.0037	0.0033	0.0050	0.0050	0.0050	0.0050	0.0048	0.0049	0.0036	0.0048	0.0050	0.0043	0.0044	0.0047
**8**	14	0.0035	0.0038	0.0045	0.0049	0.0047	0.0046	0.0045	0.0046	0.0048	0.0035	0.0049	0.0050	0.0045	0.0043	0.0044
**8**	15	0.0041	0.0043	0.0042	0.0045	0.0043	0.0045	0.0043	0.0048	0.0048	0.0037	0.0046	0.0047	0.0046	0.0045	0.0048
**9**	1	0.0035	0.0032	0.0046	0.0046	0.0046	0.0046	0.0046	0.0042	0.0049	0.0041	0.0046	0.0046	0.0049	0.0049	0.0051
**9**	2	0.0038	0.0038	0.0036	0.0048	0.0046	0.0042	0.0042	0.0041	0.0054	0.0044	0.0043	0.0045	0.0050	0.0050	0.0051
**9**	3	0.0047	0.0046	0.0042	0.0048	0.0048	0.0051	0.0051	0.0041	0.0049	0.0040	0.0041	0.0042	0.0039	0.0040	0.0044
**9**	4	0.0035	0.0033	0.0042	0.0050	0.0050	0.0050	0.0050	0.0039	0.0050	0.0042	0.0043	0.0041	0.0049	0.0049	0.0044
**9**	5	0.0045	0.0044	0.0044	0.0048	0.0048	0.0046	0.0046	0.0037	0.0046	0.0037	0.0041	0.0037	0.0048	0.0050	0.0050
**9**	6	0.0042	0.0042	0.0042	0.0047	0.0047	0.0046	0.0046	0.0040	0.0047	0.0044	0.0042	0.0042	0.0044	0.0044	0.0050
**9**	7	0.0041	0.0041	0.0035	0.0050	0.0050	0.0050	0.0050	0.0041	0.0045	0.0035	0.0041	0.0041	0.0050	0.0050	0.0050
**9**	8	0.0040	0.0043	0.0042	0.0048	0.0048	0.0048	0.0048	0.0043	0.0047	0.0034	0.0044	0.0044	0.0045	0.0044	0.0047
**9**	10	0.0043	0.0046	0.0037	0.0049	0.0048	0.0050	0.0050	0.0037	0.0051	0.0026	0.0042	0.0040	0.0049	0.0049	0.0051
**9**	11	0.0035	0.0037	0.0043	0.0045	0.0045	0.0043	0.0043	0.0031	0.0052	0.0045	0.0046	0.0046	0.0051	0.0051	0.0052
**9**	12	0.0037	0.0035	0.0045	0.0039	0.0040	0.0046	0.0047	0.0044	0.0051	0.0044	0.0046	0.0046	0.0050	0.0049	0.0050
**9**	13	0.0035	0.0035	0.0031	0.0050	0.0050	0.0050	0.0050	0.0044	0.0050	0.0042	0.0044	0.0044	0.0046	0.0048	0.0048
**9**	14	0.0034	0.0036	0.0043	0.0049	0.0048	0.0047	0.0046	0.0043	0.0049	0.0040	0.0045	0.0045	0.0049	0.0048	0.0046
**9**	15	0.0040	0.0040	0.0040	0.0046	0.0043	0.0046	0.0043	0.0044	0.0049	0.0043	0.0042	0.0042	0.0049	0.0049	0.0049
**10**	1	0.0038	0.0037	0.0042	0.0046	0.0045	0.0047	0.0047	0.0039	0.0051	0.0027	0.0049	0.0046	0.0049	0.0049	0.0053
**10**	2	0.0041	0.0044	0.0033	0.0048	0.0046	0.0043	0.0043	0.0038	0.0057	0.0029	0.0046	0.0045	0.0051	0.0051	0.0054
**10**	3	0.0051	0.0053	0.0039	0.0048	0.0047	0.0052	0.0052	0.0038	0.0051	0.0026	0.0043	0.0042	0.0039	0.0041	0.0046
**10**	4	0.0038	0.0039	0.0039	0.0051	0.0050	0.0052	0.0052	0.0036	0.0052	0.0027	0.0046	0.0041	0.0049	0.0049	0.0047
**10**	5	0.0049	0.0051	0.0040	0.0049	0.0047	0.0048	0.0048	0.0035	0.0049	0.0024	0.0043	0.0037	0.0049	0.0050	0.0052
**10**	6	0.0046	0.0049	0.0039	0.0047	0.0046	0.0047	0.0047	0.0037	0.0049	0.0029	0.0045	0.0042	0.0045	0.0045	0.0053

**10**	7	0.0044	0.0047	0.0032	0.0050	0.0049	0.0051	0.0051	0.0038	0.0047	0.0022	0.0043	0.0041	0.0050	0.0050	0.0053
**10**	8	0.0044	0.0050	0.0039	0.0049	0.0047	0.0050	0.0050	0.0040	0.0050	0.0022	0.0046	0.0044	0.0046	0.0045	0.0050
**10**	9	0.0043	0.0046	0.0037	0.0049	0.0048	0.0050	0.0050	0.0037	0.0051	0.0026	0.0042	0.0040	0.0049	0.0049	0.0051
**10**	11	0.0038	0.0043	0.0040	0.0046	0.0045	0.0044	0.0044	0.0029	0.0055	0.0029	0.0049	0.0046	0.0052	0.0052	0.0055
**10**	12	0.0040	0.0041	0.0042	0.0039	0.0040	0.0047	0.0048	0.0041	0.0053	0.0029	0.0048	0.0046	0.0050	0.0049	0.0052
**10**	13	0.0038	0.0040	0.0029	0.0050	0.0049	0.0052	0.0052	0.0041	0.0053	0.0027	0.0046	0.0044	0.0047	0.0049	0.0051
**10**	14	0.0037	0.0042	0.0040	0.0050	0.0047	0.0048	0.0047	0.0040	0.0052	0.0026	0.0047	0.0045	0.0049	0.0048	0.0048
**10**	15	0.0044	0.0047	0.0037	0.0046	0.0043	0.0047	0.0045	0.0041	0.0052	0.0028	0.0045	0.0042	0.0050	0.0050	0.0052
**11**	1	0.0031	0.0029	0.0050	0.0043	0.0043	0.0040	0.0040	0.0033	0.0052	0.0047	0.0053	0.0053	0.0051	0.0051	0.0054
**11**	2	0.0033	0.0035	0.0039	0.0045	0.0043	0.0036	0.0036	0.0032	0.0058	0.0051	0.0050	0.0053	0.0053	0.0053	0.0055
**11**	3	0.0041	0.0043	0.0046	0.0044	0.0044	0.0044	0.0044	0.0032	0.0052	0.0045	0.0047	0.0049	0.0041	0.0043	0.0047
**11**	4	0.0031	0.0031	0.0046	0.0047	0.0047	0.0044	0.0044	0.0031	0.0053	0.0048	0.0050	0.0048	0.0051	0.0051	0.0048
**11**	5	0.0040	0.0040	0.0047	0.0045	0.0045	0.0040	0.0040	0.0029	0.0049	0.0043	0.0047	0.0044	0.0051	0.0052	0.0053
**11**	6	0.0037	0.0039	0.0046	0.0044	0.0044	0.0040	0.0040	0.0031	0.0050	0.0051	0.0049	0.0049	0.0047	0.0047	0.0054
**11**	7	0.0036	0.0038	0.0038	0.0047	0.0047	0.0044	0.0044	0.0032	0.0048	0.0040	0.0048	0.0048	0.0053	0.0053	0.0054
**11**	8	0.0035	0.0040	0.0046	0.0045	0.0045	0.0042	0.0042	0.0034	0.0051	0.0039	0.0051	0.0052	0.0048	0.0047	0.0051
**11**	9	0.0035	0.0037	0.0043	0.0045	0.0045	0.0043	0.0043	0.0031	0.0052	0.0045	0.0046	0.0046	0.0051	0.0051	0.0052
**11**	10	0.0038	0.0043	0.0040	0.0046	0.0045	0.0044	0.0044	0.0029	0.0055	0.0029	0.0049	0.0046	0.0052	0.0052	0.0055
**11**	12	0.0032	0.0033	0.0049	0.0036	0.0038	0.0040	0.0041	0.0035	0.0054	0.0050	0.0053	0.0053	0.0052	0.0051	0.0053
**11**	13	0.0031	0.0032	0.0034	0.0047	0.0047	0.0044	0.0044	0.0035	0.0054	0.0048	0.0051	0.0052	0.0049	0.0051	0.0052
**11**	14	0.0030	0.0033	0.0047	0.0046	0.0045	0.0041	0.0040	0.0034	0.0053	0.0046	0.0052	0.0052	0.0051	0.0050	0.0049
**11**	15	0.0036	0.0037	0.0044	0.0043	0.0040	0.0040	0.0038	0.0035	0.0053	0.0049	0.0049	0.0049	0.0052	0.0052	0.0053
**12**	1	0.0032	0.0028	0.0052	0.0036	0.0038	0.0043	0.0044	0.0047	0.0051	0.0046	0.0052	0.0052	0.0050	0.0049	0.0052
**12**	2	0.0035	0.0033	0.0041	0.0038	0.0039	0.0039	0.0040	0.0045	0.0056	0.0049	0.0050	0.0052	0.0051	0.0050	0.0052
**12**	3	0.0043	0.0041	0.0047	0.0038	0.0040	0.0048	0.0049	0.0045	0.0051	0.0044	0.0047	0.0048	0.0039	0.0041	0.0045
**12**	4	0.0032	0.0029	0.0048	0.0040	0.0042	0.0047	0.0048	0.0043	0.0051	0.0047	0.0049	0.0047	0.0050	0.0049	0.0045
**12**	5	0.0041	0.0038	0.0049	0.0038	0.0040	0.0043	0.0044	0.0041	0.0048	0.0041	0.0047	0.0043	0.0049	0.0050	0.0051
**12**	6	0.0039	0.0037	0.0048	0.0037	0.0039	0.0043	0.0044	0.0044	0.0049	0.0049	0.0049	0.0049	0.0045	0.0044	0.0051
**12**	7	0.0038	0.0036	0.0039	0.0040	0.0042	0.0047	0.0048	0.0045	0.0047	0.0038	0.0047	0.0047	0.0051	0.0050	0.0051
**12**	8	0.0037	0.0038	0.0047	0.0038	0.0040	0.0045	0.0046	0.0047	0.0049	0.0038	0.0050	0.0051	0.0046	0.0044	0.0048

**12**	9	0.0037	0.0035	0.0045	0.0039	0.0040	0.0046	0.0047	0.0044	0.0051	0.0044	0.0046	0.0046	0.0050	0.0049	0.0050
**12**	10	0.0040	0.0041	0.0042	0.0039	0.0040	0.0047	0.0048	0.0041	0.0053	0.0029	0.0048	0.0046	0.0050	0.0049	0.0052
**12**	11	0.0032	0.0033	0.0049	0.0036	0.0038	0.0040	0.0041	0.0035	0.0054	0.0050	0.0053	0.0053	0.0052	0.0051	0.0053
**12**	13	0.0032	0.0031	0.0035	0.0040	0.0042	0.0047	0.0048	0.0049	0.0052	0.0046	0.0050	0.0051	0.0047	0.0048	0.0049
**12**	14	0.0032	0.0032	0.0049	0.0039	0.0040	0.0044	0.0044	0.0048	0.0051	0.0045	0.0051	0.0051	0.0050	0.0048	0.0047
**12**	15	0.0037	0.0035	0.0045	0.0036	0.0036	0.0043	0.0042	0.0049	0.0051	0.0048	0.0048	0.0048	0.0050	0.0049	0.0050
**13**	1	0.0031	0.0028	0.0036	0.0047	0.0047	0.0047	0.0047	0.0047	0.0050	0.0043	0.0050	0.0051	0.0046	0.0048	0.0050
**13**	2	0.0033	0.0033	0.0028	0.0049	0.0048	0.0043	0.0043	0.0045	0.0056	0.0047	0.0047	0.0051	0.0048	0.0050	0.0051
**13**	3	0.0041	0.0040	0.0033	0.0049	0.0049	0.0052	0.0052	0.0046	0.0050	0.0042	0.0045	0.0047	0.0037	0.0040	0.0043
**13**	4	0.0031	0.0029	0.0033	0.0052	0.0052	0.0052	0.0052	0.0044	0.0051	0.0044	0.0047	0.0046	0.0046	0.0048	0.0044
**13**	5	0.0039	0.0038	0.0034	0.0049	0.0049	0.0047	0.0047	0.0042	0.0047	0.0039	0.0045	0.0042	0.0046	0.0049	0.0049
**13**	6	0.0037	0.0037	0.0033	0.0048	0.0048	0.0047	0.0047	0.0045	0.0048	0.0047	0.0046	0.0047	0.0042	0.0044	0.0050
**13**	7	0.0036	0.0036	0.0027	0.0051	0.0051	0.0051	0.0051	0.0046	0.0046	0.0037	0.0045	0.0046	0.0048	0.0050	0.0050
**13**	8	0.0035	0.0037	0.0033	0.0050	0.0050	0.0050	0.0050	0.0048	0.0049	0.0036	0.0048	0.0050	0.0043	0.0044	0.0047
**13**	9	0.0035	0.0035	0.0031	0.0050	0.0050	0.0050	0.0050	0.0044	0.0050	0.0042	0.0044	0.0044	0.0046	0.0048	0.0048
**13**	10	0.0038	0.0040	0.0029	0.0050	0.0049	0.0052	0.0052	0.0041	0.0053	0.0027	0.0046	0.0044	0.0047	0.0049	0.0051
**13**	11	0.0031	0.0032	0.0034	0.0047	0.0047	0.0044	0.0044	0.0035	0.0054	0.0048	0.0051	0.0052	0.0049	0.0051	0.0052
**13**	12	0.0032	0.0031	0.0035	0.0040	0.0042	0.0047	0.0048	0.0049	0.0052	0.0046	0.0050	0.0051	0.0047	0.0048	0.0049
**13**	14	0.0030	0.0031	0.0034	0.0051	0.0049	0.0048	0.0047	0.0048	0.0051	0.0043	0.0049	0.0050	0.0046	0.0047	0.0045
**13**	15	0.0035	0.0035	0.0032	0.0047	0.0045	0.0047	0.0045	0.0050	0.0051	0.0045	0.0046	0.0047	0.0047	0.0049	0.0049
**14**	1	0.0030	0.0029	0.0050	0.0046	0.0045	0.0044	0.0043	0.0046	0.0050	0.0042	0.0051	0.0051	0.0049	0.0048	0.0048
**14**	2	0.0032	0.0034	0.0039	0.0048	0.0045	0.0040	0.0039	0.0044	0.0055	0.0045	0.0048	0.0051	0.0050	0.0049	0.0048
**14**	3	0.0040	0.0041	0.0045	0.0048	0.0047	0.0049	0.0048	0.0044	0.0050	0.0040	0.0046	0.0047	0.0039	0.0040	0.0041
**14**	4	0.0030	0.0030	0.0046	0.0051	0.0049	0.0048	0.0047	0.0042	0.0050	0.0043	0.0048	0.0046	0.0049	0.0048	0.0042
**14**	5	0.0039	0.0039	0.0047	0.0049	0.0047	0.0044	0.0043	0.0041	0.0047	0.0038	0.0046	0.0042	0.0048	0.0049	0.0047
**14**	6	0.0037	0.0038	0.0046	0.0048	0.0046	0.0044	0.0043	0.0043	0.0048	0.0045	0.0048	0.0048	0.0044	0.0043	0.0047
**14**	7	0.0035	0.0037	0.0038	0.0051	0.0049	0.0048	0.0047	0.0044	0.0046	0.0035	0.0046	0.0046	0.0050	0.0049	0.0047
**14**	8	0.0035	0.0038	0.0045	0.0049	0.0047	0.0046	0.0045	0.0046	0.0048	0.0035	0.0049	0.0050	0.0045	0.0043	0.0044
**14**	9	0.0034	0.0036	0.0043	0.0049	0.0048	0.0047	0.0046	0.0043	0.0049	0.0040	0.0045	0.0045	0.0049	0.0048	0.0046
**14**	10	0.0037	0.0042	0.0040	0.0050	0.0047	0.0048	0.0047	0.0040	0.0052	0.0026	0.0047	0.0045	0.0049	0.0048	0.0048

**14**	11	0.0030	0.0033	0.0047	0.0046	0.0045	0.0041	0.0040	0.0034	0.0053	0.0046	0.0052	0.0052	0.0051	0.0050	0.0049
**14**	12	0.0032	0.0032	0.0049	0.0039	0.0040	0.0044	0.0044	0.0048	0.0051	0.0045	0.0051	0.0051	0.0050	0.0048	0.0047
**14**	13	0.0030	0.0031	0.0034	0.0051	0.0049	0.0048	0.0047	0.0048	0.0051	0.0043	0.0049	0.0050	0.0046	0.0047	0.0045
**14**	15	0.0035	0.0036	0.0044	0.0046	0.0042	0.0044	0.0041	0.0048	0.0050	0.0044	0.0047	0.0047	0.0049	0.0048	0.0046
**15**	1	0.0035	0.0032	0.0046	0.0043	0.0041	0.0043	0.0041	0.0047	0.0049	0.0045	0.0048	0.0048	0.0049	0.0049	0.0051
**15**	2	0.0038	0.0038	0.0036	0.0045	0.0041	0.0039	0.0037	0.0045	0.0055	0.0048	0.0046	0.0048	0.0051	0.0051	0.0052
**15**	3	0.0047	0.0046	0.0042	0.0045	0.0042	0.0048	0.0045	0.0046	0.0049	0.0043	0.0043	0.0044	0.0039	0.0041	0.0044
**15**	4	0.0035	0.0034	0.0043	0.0047	0.0045	0.0047	0.0045	0.0044	0.0050	0.0045	0.0046	0.0044	0.0049	0.0049	0.0045
**15**	5	0.0046	0.0044	0.0044	0.0045	0.0043	0.0043	0.0041	0.0042	0.0047	0.0040	0.0043	0.0040	0.0049	0.0050	0.0050
**15**	6	0.0043	0.0043	0.0043	0.0044	0.0042	0.0043	0.0041	0.0045	0.0047	0.0048	0.0045	0.0045	0.0045	0.0045	0.0050
**15**	7	0.0041	0.0041	0.0035	0.0047	0.0044	0.0047	0.0044	0.0046	0.0046	0.0037	0.0043	0.0043	0.0051	0.0051	0.0051
**15**	8	0.0041	0.0043	0.0042	0.0045	0.0043	0.0045	0.0043	0.0048	0.0048	0.0037	0.0046	0.0047	0.0046	0.0045	0.0048
**15**	9	0.0040	0.0040	0.0040	0.0046	0.0043	0.0046	0.0043	0.0044	0.0049	0.0043	0.0042	0.0042	0.0049	0.0049	0.0049
**15**	10	0.0044	0.0047	0.0037	0.0046	0.0043	0.0047	0.0045	0.0041	0.0052	0.0028	0.0045	0.0042	0.0050	0.0050	0.0052
**15**	11	0.0036	0.0037	0.0044	0.0043	0.0040	0.0040	0.0038	0.0035	0.0053	0.0049	0.0049	0.0049	0.0052	0.0052	0.0053
**15**	12	0.0037	0.0035	0.0045	0.0036	0.0036	0.0043	0.0042	0.0049	0.0051	0.0048	0.0048	0.0048	0.0050	0.0049	0.0050
**15**	13	0.0035	0.0035	0.0032	0.0047	0.0045	0.0047	0.0045	0.0050	0.0051	0.0045	0.0046	0.0047	0.0047	0.0049	0.0049
**15**	14	0.0035	0.0036	0.0044	0.0046	0.0042	0.0044	0.0041	0.0048	0.0050	0.0044	0.0047	0.0047	0.0049	0.0048	0.0046

Table S7 Linear normalization (cost & benefit criterion) for denim jacket using Equation 3 and 4

	**C1**	**C2**	**C3**	**C4**	**C5**	**C6**	**C7**	**C8**	**C9**	**C10**	**C11**	**C12**	**C13**	**C14**	**C15**
**1**	0.4677	0.2611	0.4458	0.2876	0.0000	0.1554	0.0000	0.0000	0.3488	1.0000	1.0000	0.2500	0.5000	0.7895	0.0000
**2**	0.3781	0.3788	0.3503	0.4378	0.0609	0.3801	0.3929	1.0000	0.2171	0.5098	0.7500	0.0000	0.9167	0.8947	0.0000
**3**	0.3653	0.4210	0.4214	0.4371	0.1254	0.4929	0.4205	1.0000	0.1550	0.1321	0.7500	0.0000	0.8333	0.9474	0.0000
**4**	0.0237	0.0000	0.5210	0.3205	0.0838	0.3684	0.3302	0.0000	1.0000	0.9389	0.7500	0.0000	0.9167	0.7895	0.0000
**5**	0.4059	0.4736	0.9770	0.7532	0.1106	0.3817	0.5099	0.0000	0.2093	0.3454	1.0000	0.5000	0.4167	0.7895	1.0000
**6**	0.6987	0.6897	0.5603	0.2647	0.0543	0.3548	0.1312	0.0000	0.1705	0.6752	0.0000	0.0000	1.0000	0.5789	0.0000
**7**	0.8398	0.6420	0.5881	0.3813	0.0817	0.2818	0.2267	1.0000	0.1860	0.5649	1.0000	0.7500	0.6667	0.0000	0.0000
**8**	0.2331	0.3519	0.9560	0.8011	0.0591	0.4309	0.2680	1.0000	0.2093	0.2030	1.0000	0.5000	1.0000	0.5789	0.0000

**9**	0.4977	0.4577	0.2473	0.4006	1.0000	0.2993	0.4214	1.0000	0.1860	0.2499	1.0000	0.5000	0.6667	0.3158	0.0000
**10**	0.0000	0.1837	0.7304	0.5994	0.2053	0.4693	0.9432	0.0000	0.5581	0.2336	1.0000	0.0000	0.3333	0.7895	0.0000
**11**	0.4021	0.6686	0.0000	0.6688	0.2469	0.7288	0.5144	0.0000	0.3876	0.0000	0.5000	0.0000	1.0000	1.0000	0.0000
**12**	0.6790	0.6438	0.7981	1.0000	0.3955	0.7681	0.3762	0.0000	0.0000	0.5199	1.0000	0.7500	0.7500	0.6842	0.0000
**13**	0.7105	0.5870	1.0000	0.0000	1.0000	0.0000	0.2465	1.0000	0.2171	0.4871	0.5000	0.0000	0.9167	0.9211	0.0000
**14**	0.1273	0.4995	0.7622	0.7361	0.2674	1.0000	1.0000	1.0000	0.4264	0.0316	0.7500	0.5000	0.0000	0.3684	0.0000
**15**	1.0000	1.0000	0.6944	0.2332	1.0000	0.1042	0.4823	0.0000	0.0698	0.2576	1.0000	1.0000	0.7083	0.4737	0.0000
**16**	0.3554	0.2635	0.5129	0.5322	0.0235	0.3303	0.2234	1.0000	0.0155	0.9038	0.2500	0.0000	0.8333	1.0000	0.0000

Table S8 Vector normalization (cost & benefit criterion) for denim jacket using Equation 5 and 6

	**C1**	**C2**	**C3**	**C4**	**C5**	**C6**	**C7**	**C8**	**C9**	**C10**	**C11**	**C12**	**C13**	**C14**	**C15**
**1**	0.9518	0.9260	0.9760	0.8223	0.6932	0.7857	0.8477	0.9403	0.6985	1.0000	1.0000	0.8009	0.6826	0.8741	0.9734
**2**	0.9437	0.9378	0.9719	0.8597	0.7119	0.8427	0.9075	1.0000	0.6375	0.8644	0.9704	0.7345	0.9471	0.9370	0.9734
**3**	0.9426	0.9420	0.9749	0.8596	0.7317	0.8713	0.9117	1.0000	0.6088	0.7599	0.9704	0.7345	0.8942	0.9685	0.9734
**4**	0.9117	0.8999	0.9792	0.8305	0.7189	0.8397	0.8980	0.9403	1.0000	0.9831	0.9704	0.7345	0.9471	0.8741	0.9734
**5**	0.9462	0.9473	0.9990	0.9384	0.7271	0.8431	0.9254	0.9403	0.6339	0.8189	1.0000	0.8673	0.6297	0.8741	1.0000
**6**	0.9727	0.9689	0.9809	0.8165	0.7098	0.8363	0.8677	0.9403	0.6160	0.9101	0.8817	0.7345	1.0000	0.7482	0.9734
**7**	0.9855	0.9641	0.9822	0.8456	0.7182	0.8177	0.8822	1.0000	0.6231	0.8796	1.0000	0.9336	0.7884	0.4019	0.9734
**8**	0.9306	0.9351	0.9981	0.9504	0.7113	0.8556	0.8885	1.0000	0.6339	0.7795	1.0000	0.8673	1.0000	0.7482	0.9734
**9**	0.9545	0.9457	0.9674	0.8505	1.0000	0.8222	0.9119	1.0000	0.6231	0.7925	1.0000	0.8673	0.7884	0.5908	0.9734
**10**	0.9095	0.9183	0.9883	0.9001	0.7562	0.8653	0.9913	0.9403	0.7954	0.7880	1.0000	0.7345	0.5767	0.8741	0.9734
**11**	0.9459	0.9668	0.9567	0.9174	0.7689	0.9312	0.9260	0.9403	0.7165	0.7233	0.9409	0.7345	1.0000	1.0000	0.9734
**12**	0.9710	0.9643	0.9913	1.0000	0.8145	0.9412	0.9050	0.9403	0.5370	0.8672	1.0000	0.9336	0.8413	0.8111	0.9734
**13**	0.9738	0.9586	1.0000	0.7505	1.0000	0.7462	0.8852	1.0000	0.6375	0.8581	0.9409	0.7345	0.9471	0.9528	0.9734
**14**	0.9210	0.9499	0.9897	0.9341	0.7752	1.0000	1.0000	1.0000	0.7344	0.7321	0.9704	0.8673	0.3651	0.6223	0.9734
**15**	1.0000	1.0000	0.9868	0.8087	1.0000	0.7727	0.9211	0.9403	0.5693	0.7946	1.0000	1.0000	0.8148	0.6852	0.9734
**16**	0.9417	0.9262	0.9789	0.8833	0.7004	0.8301	0.8817	1.0000	0.5442	0.9734	0.9113	0.7345	0.8942	1.0000	0.9734

Table S9 Calculation of $w_j\left(1 - x_{ij}^{1}\right)$ for denim jacket

	**C1**	**C2**	**C3**	**C4**	**C5**	**C6**	**C7**	**C8**	**C9**	**C10**	**C11**	**C12**	**C13**	**C14**	**C15**

**1**	0.0338	0.0460	0.0351	0.0479	0.0667	0.0560	0.0662	0.0660	0.0446	0.0000	0.0000	0.0499	0.0349	0.0147	0.0708
**2**	0.0395	0.0387	0.0411	0.0378	0.0626	0.0411	0.0402	0.0000	0.0536	0.0324	0.0168	0.0665	0.0058	0.0073	0.0708
**3**	0.0403	0.0360	0.0366	0.0378	0.0583	0.0336	0.0384	0.0000	0.0578	0.0574	0.0168	0.0665	0.0116	0.0037	0.0708
**4**	0.0620	0.0622	0.0303	0.0457	0.0611	0.0419	0.0443	0.0660	0.0000	0.0040	0.0168	0.0665	0.0058	0.0147	0.0708
**5**	0.0377	0.0328	0.0015	0.0166	0.0593	0.0410	0.0324	0.0660	0.0541	0.0433	0.0000	0.0332	0.0407	0.0147	0.0000
**6**	0.0191	0.0193	0.0278	0.0494	0.0630	0.0428	0.0575	0.0660	0.0568	0.0215	0.0672	0.0665	0.0000	0.0293	0.0708
**7**	0.0102	0.0223	0.0261	0.0416	0.0612	0.0476	0.0512	0.0000	0.0557	0.0288	0.0000	0.0166	0.0233	0.0697	0.0708
**8**	0.0487	0.0403	0.0028	0.0134	0.0627	0.0377	0.0484	0.0000	0.0541	0.0527	0.0000	0.0332	0.0000	0.0293	0.0708
**9**	0.0319	0.0337	0.0476	0.0403	0.0000	0.0465	0.0383	0.0000	0.0557	0.0496	0.0000	0.0332	0.0233	0.0477	0.0708
**10**	0.0635	0.0508	0.0171	0.0269	0.0530	0.0352	0.0038	0.0660	0.0303	0.0506	0.0000	0.0665	0.0465	0.0147	0.0708
**11**	0.0380	0.0206	0.0633	0.0223	0.0502	0.0180	0.0321	0.0660	0.0419	0.0661	0.0336	0.0665	0.0000	0.0000	0.0708
**12**	0.0204	0.0222	0.0128	0.0000	0.0403	0.0154	0.0413	0.0660	0.0685	0.0317	0.0000	0.0166	0.0174	0.0220	0.0708
**13**	0.0184	0.0257	0.0000	0.0672	0.0000	0.0663	0.0499	0.0000	0.0536	0.0339	0.0336	0.0665	0.0058	0.0055	0.0708
**14**	0.0554	0.0311	0.0150	0.0177	0.0488	0.0000	0.0000	0.0000	0.0393	0.0640	0.0168	0.0332	0.0698	0.0440	0.0708
**15**	0.0000	0.0000	0.0193	0.0515	0.0000	0.0594	0.0343	0.0660	0.0637	0.0491	0.0000	0.0000	0.0204	0.0367	0.0708
**16**	0.0409	0.0458	0.0308	0.0314	0.0651	0.0444	0.0514	0.0000	0.0674	0.0064	0.0504	0.0665	0.0116	0.0000	0.0708

Table S10 Calculation of $\left[x_{ij}^{2\,w_j}\right]$ for denim jacket

	**C1**	**C2**	**C3**	**C4**	**C5**	**C6**	**C7**	**C8**	**C9**	**C10**	**C11**	**C12**	**C13**	**C14**	**C15**
**1**	0.9969	0.9952	0.9985	0.9869	0.9759	0.9841	0.9891	0.9959	0.9757	1.0000	1.0000	0.9853	0.9737	0.9907	0.9981
**2**	0.9963	0.9960	0.9982	0.9899	0.9776	0.9887	0.9936	1.0000	0.9696	0.9904	0.9980	0.9797	0.9962	0.9955	0.9981
**3**	0.9963	0.9963	0.9984	0.9899	0.9794	0.9909	0.9939	1.0000	0.9666	0.9820	0.9980	0.9797	0.9922	0.9978	0.9981
**4**	0.9941	0.9935	0.9987	0.9876	0.9782	0.9885	0.9929	0.9959	1.0000	0.9989	0.9980	0.9797	0.9962	0.9907	0.9981
**5**	0.9965	0.9966	0.9999	0.9957	0.9790	0.9887	0.9949	0.9959	0.9693	0.9869	1.0000	0.9906	0.9682	0.9907	1.0000
**6**	0.9982	0.9980	0.9988	0.9865	0.9774	0.9882	0.9907	0.9959	0.9674	0.9938	0.9916	0.9797	1.0000	0.9800	0.9981
**7**	0.9991	0.9977	0.9989	0.9888	0.9782	0.9867	0.9917	1.0000	0.9681	0.9916	1.0000	0.9954	0.9835	0.9385	0.9981
**8**	0.9954	0.9958	0.9999	0.9966	0.9775	0.9897	0.9922	1.0000	0.9693	0.9837	1.0000	0.9906	1.0000	0.9800	0.9981
**9**	0.9971	0.9965	0.9979	0.9892	1.0000	0.9871	0.9939	1.0000	0.9681	0.9847	1.0000	0.9906	0.9835	0.9640	0.9981
**10**	0.9940	0.9947	0.9993	0.9929	0.9815	0.9905	0.9994	0.9959	0.9845	0.9844	1.0000	0.9797	0.9623	0.9907	0.9981

**11**	0.9965	0.9979	0.9972	0.9942	0.9826	0.9953	0.9949	0.9959	0.9774	0.9788	0.9959	0.9797	1.0000	1.0000	0.9981
**12**	0.9981	0.9977	0.9994	1.0000	0.9864	0.9960	0.9934	0.9959	0.9583	0.9906	1.0000	0.9954	0.9880	0.9855	0.9981
**13**	0.9983	0.9974	1.0000	0.9809	1.0000	0.9808	0.9920	1.0000	0.9696	0.9899	0.9959	0.9797	0.9962	0.9966	0.9981
**14**	0.9948	0.9968	0.9993	0.9954	0.9832	1.0000	1.0000	1.0000	0.9791	0.9796	0.9980	0.9906	0.9321	0.9675	0.9981
**15**	1.0000	1.0000	0.9992	0.9858	1.0000	0.9830	0.9946	0.9959	0.9622	0.9849	1.0000	1.0000	0.9858	0.9740	0.9981
**16**	0.9962	0.9952	0.9987	0.9917	0.9765	0.9877	0.9917	1.0000	0.9592	0.9982	0.9938	0.9797	0.9922	1.0000	0.9981

Table S11 Ranking results obtained using Equationf 7 to 13 for denim jacket

	$u_1(a_i)$	$u_2(a_i)$	$u_3(a_i)$		$u_1^N(a_i)$	$u_2^N(a_i)$	$u_3^N(a_i)$		$r_1(a_i)$	$r_2(a_i)$	$r_3(a_i)$		S_i	**Rank**
**1**	0.3674	0.0708	0.8562	**1**	0.1997	0.2510	0.2470	**1**	15.0000	9.0000	13.0000	**1**	0.1339	15
**2**	0.4457	0.0708	0.8752	**2**	0.2422	0.2510	0.2525	**2**	9.0000	9.0000	6.0000	**2**	0.1802	8
**3**	0.4342	0.0708	0.8677	**3**	0.2360	0.2510	0.2503	**3**	10.0000	9.0000	8.0000	**3**	0.1703	12
**4**	0.4077	0.0708	0.8961	**4**	0.2216	0.2510	0.2585	**4**	13.0000	9.0000	1.0000	**4**	0.1754	9
**5**	0.5265	0.0660	0.8620	**5**	0.2862	0.2342	0.2487	**5**	3.0000	1.0000	10.0000	**5**	0.2289	2
**6**	0.3428	0.0708	0.8545	**6**	0.1863	0.2510	0.2465	**6**	16.0000	9.0000	14.0000	**6**	0.1233	16
**7**	0.4749	0.0708	0.8297	**7**	0.2581	0.2510	0.2394	**7**	8.0000	9.0000	15.0000	**7**	0.1744	10
**8**	0.5056	0.0708	0.8760	**8**	0.2748	0.2510	0.2527	**8**	4.0000	9.0000	5.0000	**8**	0.2114	5
**9**	0.4812	0.0708	0.8599	**9**	0.2616	0.2510	0.2481	**9**	7.0000	9.0000	11.0000	**9**	0.1867	6
**10**	0.4042	0.0708	0.8576	**10**	0.2197	0.2510	0.2474	**10**	14.0000	9.0000	12.0000	**10**	0.1495	14
**11**	0.4105	0.0708	0.8901	**11**	0.2231	0.2510	0.2568	**11**	12.0000	9.0000	2.0000	**11**	0.1740	11
**12**	0.5545	0.0708	0.8886	**12**	0.3014	0.2510	0.2564	**12**	1.0000	9.0000	3.0000	**12**	0.2405	1
**13**	0.5027	0.0708	0.8819	**13**	0.2732	0.2510	0.2544	**13**	5.0000	9.0000	4.0000	**13**	0.2116	4
**14**	0.4937	0.0708	0.8277	**14**	0.2684	0.2510	0.2388	**14**	6.0000	9.0000	16.0000	**14**	0.1836	7
**15**	0.5287	0.0708	0.8711	**15**	0.2874	0.2510	0.2513	**15**	2.0000	9.0000	7.0000	**15**	0.2192	3
**16**	0.4169	0.0708	0.8671	**16**	0.2266	0.2510	0.2502	**16**	11.0000	9.0000	9.0000	**16**	0.1614	13

Table S12 Linear normalization (cost & benefit criterion) for denim pant using Equation 3 and 4

	C1	C2	C3	C4	C5	C6	C7	C8	C9	C10	C11	C12	C13	C14	C15
**1**	403.8800	0.8663	0.2837	32.6667	39.4333	390.6400	1040.9880	3.5000	203.3333	86.6500	4.5000	1.5000	3.0000	2.0000	4.5000
**2**	417.4000	0.8278	0.2884	39.6667	46.1667	530.6780	1404.0120	4.5000	146.6667	54.9700	4.0000	1.0000	0.5000	1.0000	4.5000
**3**	419.3200	0.8140	0.2849	39.6333	53.3000	600.9820	1429.5240	4.5000	120.0000	30.5600	4.0000	1.0000	1.0000	0.5000	4.5000
**4**	470.8400	0.9517	0.2800	34.2000	48.7000	523.3840	1346.0260	3.5000	483.3333	82.7000	4.0000	1.0000	0.5000	2.0000	4.5000
**5**	413.2000	0.7968	0.2576	54.3667	51.6667	531.7000	1512.0500	3.5000	143.3333	44.3400	4.5000	2.0000	3.5000	2.0000	5.0000
**6**	369.0400	0.7261	0.2781	31.6000	45.4333	514.9300	1162.2080	3.5000	126.6667	65.6600	2.5000	1.0000	0.0000	4.0000	4.5000
**7**	347.7600	0.7417	0.2767	37.0333	48.4667	469.4220	1250.4480	4.5000	133.3333	58.5300	4.5000	2.5000	2.0000	9.5000	4.5000
**8**	439.2600	0.8366	0.2586	56.6000	45.9667	562.3420	1288.5640	4.5000	143.3333	35.1400	4.5000	2.0000	0.0000	4.0000	4.5000
**9**	399.3600	0.8020	0.2935	37.9333	150.0000	480.3280	1430.2740	4.5000	133.3333	38.1700	4.5000	2.0000	2.0000	6.5000	4.5000
**10**	474.4200	0.8916	0.2697	47.2000	62.1333	586.3100	1912.3460	3.5000	293.3333	37.1200	4.5000	1.0000	4.0000	2.0000	4.5000
**11**	413.7800	0.7330	0.3057	50.4333	66.7333	748.0180	1516.1920	3.5000	220.0000	22.0200	3.5000	1.0000	0.0000	0.0000	4.5000
**12**	372.0200	0.7411	0.2664	65.8667	83.1667	772.5320	1388.5420	3.5000	53.3333	55.6200	4.5000	2.5000	1.5000	3.0000	4.5000
**13**	367.2600	0.7597	0.2565	19.2667	150.0000	293.8200	1268.7480	4.5000	146.6667	53.5000	3.5000	1.0000	0.5000	0.7500	4.5000
**14**	455.2200	0.7883	0.2682	53.5667	69.0000	917.0300	1964.8660	4.5000	236.6667	24.0600	4.0000	2.0000	6.0000	6.0000	4.5000
**15**	323.6000	0.6246	0.2715	30.1333	150.0000	358.7600	1486.5460	3.5000	83.3333	38.6700	4.5000	3.0000	1.7500	5.0000	4.5000
**16**	420.8200	0.8655	0.2804	44.0667	42.0333	499.6860	1247.4120	4.5000	60.0000	80.4300	3.0000	1.0000	1.0000	0.0000	4.5000

Table S13 Vector normalization (cost & benefit criterion) for denim pant using Equation 5 and 6

	C1	C2	C3	C4	C5	C6	C7	C8	C9	C10	C11	C12	C13	C14	C15
**1**	0.9518	0.9260	0.9760	0.8223	0.6932	0.7857	0.8477	0.9403	0.6985	1.0000	1.0000	0.8009	0.6826	0.8741	0.9734
**2**	0.9437	0.9378	0.9719	0.8597	0.7119	0.8427	0.9075	1.0000	0.6375	0.8644	0.9704	0.7345	0.9471	0.9370	0.9734
**3**	0.9426	0.9420	0.9749	0.8596	0.7317	0.8713	0.9117	1.0000	0.6088	0.7599	0.9704	0.7345	0.8942	0.9685	0.9734

**4**	0.9117	0.8999	0.9792	0.8305	0.7189	0.8397	0.8980	0.9403	1.0000	0.9831	0.9704	0.7345	0.9471	0.8741	0.9734
**5**	0.9462	0.9473	0.9990	0.9384	0.7271	0.8431	0.9254	0.9403	0.6339	0.8189	1.0000	0.8673	0.6297	0.8741	1.0000
**6**	0.9727	0.9689	0.9809	0.8165	0.7098	0.8363	0.8677	0.9403	0.6160	0.9101	0.8817	0.7345	1.0000	0.7482	0.9734
**7**	0.9855	0.9641	0.9822	0.8456	0.7182	0.8177	0.8822	1.0000	0.6231	0.8796	1.0000	0.9336	0.7884	0.4019	0.9734
**8**	0.9306	0.9351	0.9981	0.9504	0.7113	0.8556	0.8885	1.0000	0.6339	0.7795	1.0000	0.8673	1.0000	0.7482	0.9734
**9**	0.9545	0.9457	0.9674	0.8505	1.0000	0.8222	0.9119	1.0000	0.6231	0.7925	1.0000	0.8673	0.7884	0.5908	0.9734
**10**	0.9095	0.9183	0.9883	0.9001	0.7562	0.8653	0.9913	0.9403	0.7954	0.7880	1.0000	0.7345	0.5767	0.8741	0.9734
**11**	0.9459	0.9668	0.9567	0.9174	0.7689	0.9312	0.9260	0.9403	0.7165	0.7233	0.9409	0.7345	1.0000	1.0000	0.9734
**12**	0.9710	0.9643	0.9913	1.0000	0.8145	0.9412	0.9050	0.9403	0.5370	0.8672	1.0000	0.9336	0.8413	0.8111	0.9734
**13**	0.9738	0.9586	1.0000	0.7505	1.0000	0.7462	0.8852	1.0000	0.6375	0.8581	0.9409	0.7345	0.9471	0.9528	0.9734
**14**	0.9210	0.9499	0.9897	0.9341	0.7752	1.0000	1.0000	1.0000	0.7344	0.7321	0.9704	0.8673	0.3651	0.6223	0.9734
**15**	1.0000	1.0000	0.9868	0.8087	1.0000	0.7727	0.9211	0.9403	0.5693	0.7946	1.0000	1.0000	0.8148	0.6852	0.9734
**16**	0.9417	0.9262	0.9789	0.8833	0.7004	0.8301	0.8817	1.0000	0.5442	0.9734	0.9113	0.7345	0.8942	1.0000	0.9734

Table S14 Calculation of $w_j\left(1 - x_{ij}^1\right)$ for denim pant

	**C1**	**C2**	**C3**	**C4**	**C5**	**C6**	**C7**	**C8**	**C9**	**C10**	**C11**	**C12**	**C13**	**C14**	**C15**
**1**	0.0330	0.0458	0.0356	0.0484	0.0675	0.0571	0.0674	0.0642	0.0459	0.0000	0.0000	0.0509	0.0342	0.0145	0.0699
**2**	0.0386	0.0385	0.0417	0.0382	0.0634	0.0419	0.0409	0.0000	0.0552	0.0310	0.0170	0.0678	0.0057	0.0072	0.0699
**3**	0.0394	0.0359	0.0372	0.0383	0.0591	0.0343	0.0391	0.0000	0.0596	0.0548	0.0170	0.0678	0.0114	0.0036	0.0699
**4**	0.0606	0.0620	0.0308	0.0462	0.0619	0.0427	0.0452	0.0642	0.0000	0.0039	0.0170	0.0678	0.0057	0.0145	0.0699
**5**	0.0369	0.0326	0.0015	0.0168	0.0601	0.0418	0.0330	0.0642	0.0557	0.0413	0.0000	0.0339	0.0399	0.0145	0.0000
**6**	0.0187	0.0192	0.0282	0.0500	0.0639	0.0436	0.0586	0.0642	0.0585	0.0205	0.0681	0.0678	0.0000	0.0289	0.0699
**7**	0.0099	0.0222	0.0265	0.0421	0.0620	0.0486	0.0521	0.0000	0.0574	0.0275	0.0000	0.0170	0.0228	0.0687	0.0699
**8**	0.0476	0.0402	0.0028	0.0135	0.0635	0.0385	0.0494	0.0000	0.0557	0.0503	0.0000	0.0339	0.0000	0.0289	0.0699
**9**	0.0312	0.0336	0.0483	0.0408	0.0000	0.0474	0.0390	0.0000	0.0574	0.0474	0.0000	0.0339	0.0228	0.0470	0.0699
**10**	0.0620	0.0506	0.0173	0.0272	0.0537	0.0359	0.0038	0.0642	0.0312	0.0484	0.0000	0.0678	0.0456	0.0145	0.0699
**11**	0.0371	0.0205	0.0642	0.0225	0.0509	0.0183	0.0327	0.0642	0.0432	0.0632	0.0341	0.0678	0.0000	0.0000	0.0699
**12**	0.0199	0.0221	0.0130	0.0000	0.0408	0.0157	0.0421	0.0642	0.0705	0.0303	0.0000	0.0170	0.0171	0.0217	0.0699
**13**	0.0180	0.0256	0.0000	0.0680	0.0000	0.0676	0.0508	0.0000	0.0552	0.0324	0.0341	0.0678	0.0057	0.0054	0.0699
**14**	0.0541	0.0310	0.0153	0.0179	0.0495	0.0000	0.0000	0.0000	0.0404	0.0612	0.0170	0.0339	0.0685	0.0434	0.0699

**15**	0.0000	0.0000	0.0196	0.0521	0.0000	0.0606	0.0349	0.0642	0.0656	0.0469	0.0000	0.0000	0.0200	0.0362	0.0699
**16**	0.0400	0.0457	0.0313	0.0318	0.0659	0.0453	0.0524	0.0000	0.0694	0.0061	0.0511	0.0678	0.0114	0.0000	0.0699

Table S15 Calculation of $\left[x_{ij}^{2^{w_j}}\right]$ for denim pant

	**C1**	**C2**	**C3**	**C4**	**C5**	**C6**	**C7**	**C8**	**C9**	**C10**	**C11**	**C12**	**C13**	**C14**	**C15**
**1**	0.9969	0.9952	0.9984	0.9868	0.9756	0.9838	0.9889	0.9961	0.9750	1.0000	1.0000	0.9851	0.9742	0.9908	0.9981
**2**	0.9964	0.9960	0.9982	0.9898	0.9773	0.9885	0.9935	1.0000	0.9688	0.9908	0.9980	0.9793	0.9963	0.9955	0.9981
**3**	0.9963	0.9963	0.9984	0.9898	0.9791	0.9907	0.9938	1.0000	0.9656	0.9828	0.9980	0.9793	0.9924	0.9978	0.9981
**4**	0.9943	0.9935	0.9987	0.9874	0.9780	0.9883	0.9928	0.9961	1.0000	0.9989	0.9980	0.9793	0.9963	0.9908	0.9981
**5**	0.9966	0.9966	0.9999	0.9957	0.9787	0.9885	0.9948	0.9961	0.9684	0.9875	1.0000	0.9904	0.9688	0.9908	1.0000
**6**	0.9983	0.9980	0.9988	0.9863	0.9771	0.9880	0.9905	0.9961	0.9664	0.9941	0.9915	0.9793	1.0000	0.9803	0.9981
**7**	0.9991	0.9977	0.9988	0.9887	0.9779	0.9865	0.9916	1.0000	0.9672	0.9919	1.0000	0.9954	0.9838	0.9393	0.9981
**8**	0.9955	0.9958	0.9999	0.9965	0.9773	0.9895	0.9921	1.0000	0.9684	0.9844	1.0000	0.9904	1.0000	0.9803	0.9981
**9**	0.9971	0.9965	0.9979	0.9890	1.0000	0.9868	0.9938	1.0000	0.9672	0.9854	1.0000	0.9904	0.9838	0.9645	0.9981
**10**	0.9941	0.9947	0.9992	0.9929	0.9813	0.9903	0.9994	0.9961	0.9840	0.9851	1.0000	0.9793	0.9630	0.9908	0.9981
**11**	0.9966	0.9979	0.9972	0.9942	0.9824	0.9952	0.9948	0.9961	0.9768	0.9798	0.9959	0.9793	1.0000	1.0000	0.9981
**12**	0.9982	0.9978	0.9994	1.0000	0.9862	0.9959	0.9933	0.9961	0.9571	0.9910	1.0000	0.9954	0.9882	0.9857	0.9981
**13**	0.9984	0.9974	1.0000	0.9807	1.0000	0.9804	0.9918	1.0000	0.9688	0.9904	0.9959	0.9793	0.9963	0.9967	0.9981
**14**	0.9949	0.9968	0.9993	0.9954	0.9830	1.0000	1.0000	1.0000	0.9785	0.9805	0.9980	0.9904	0.9333	0.9679	0.9981
**15**	1.0000	1.0000	0.9991	0.9857	1.0000	0.9827	0.9945	0.9961	0.9611	0.9856	1.0000	1.0000	0.9861	0.9744	0.9981
**16**	0.9963	0.9953	0.9986	0.9916	0.9762	0.9875	0.9915	1.0000	0.9580	0.9983	0.9937	0.9793	0.9924	1.0000	0.9981

Table S16 Ranking results obtained using Equationf 7 to 13 for denim pant

	$u_1(a_i)$	$u_2(a_i)$	$u_3(a_i)$		$u_1^N(a_i)$	$u_2^N(a_i)$	$u_3^N(a_i)$		$r_1(a_i)$	$r_2(a_i)$	$r_3(a_i)$		S_i	**Rank**
**1**	0.3652	0.0699	0.8551	1	0.1987	0.2511	0.2469	1	15.0000	8.5000	13.0000	1	0.1337	15
**2**	0.4426	0.0699	0.8739	2	0.2408	0.2511	0.2523	2	9.0000	8.5000	6.0000	2	0.1797	8
**3**	0.4325	0.0699	0.8668	3	0.2353	0.2511	0.2502	3	10.0000	8.5000	8.0000	3	0.1704	12
**4**	0.4075	0.0699	0.8954	4	0.2217	0.2511	0.2585	4	14.0000	8.5000	1.0000	4	0.1754	9
**5**	0.5275	0.0642	0.8618	5	0.2870	0.2306	0.2488	5	3.0000	1.0000	10.0000	5	0.2321	1
**6**	0.3395	0.0699	0.8531	6	0.1848	0.2511	0.2463	6	16.0000	8.5000	14.0000	6	0.1226	16

**7**	0.4731	0.0699	0.8294	7	0.2574	0.2511	0.2395	7	8.0000	8.5000	15.0000	7	0.1746	11
**8**	0.5054	0.0699	0.8754	8	0.2750	0.2511	0.2527	8	4.0000	8.5000	5.0000	8	0.2122	4
**9**	0.4811	0.0699	0.8599	9	0.2618	0.2511	0.2482	9	7.0000	8.5000	11.0000	9	0.1876	6
**10**	0.4076	0.0699	0.8579	10	0.2218	0.2511	0.2477	10	13.0000	8.5000	12.0000	10	0.1523	14
**11**	0.4111	0.0699	0.8897	11	0.2237	0.2511	0.2569	11	12.0000	8.5000	2.0000	11	0.1750	10
**12**	0.5555	0.0705	0.8879	12	0.3023	0.2532	0.2563	12	1.0000	16.0000	3.0000	12	0.2273	2
**13**	0.4993	0.0699	0.8805	13	0.2717	0.2511	0.2542	13	5.0000	8.5000	4.0000	13	0.2111	5
**14**	0.4976	0.0699	0.8291	14	0.2708	0.2511	0.2394	14	6.0000	8.5000	16.0000	14	0.1862	7
**15**	0.5298	0.0699	0.8708	15	0.2883	0.2511	0.2514	15	2.0000	8.5000	7.0000	15	0.2205	3
**16**	0.4117	0.0699	0.8652	16	0.2240	0.2511	0.2498	16	11.0000	8.5000	9.0000	16	0.1599	13
